# A murine experimental model of the pulmonary thrombotic effect induced by the venom of the snake *Bothrops lanceolatus*

**DOI:** 10.1371/journal.pntd.0012335

**Published:** 2024-10-02

**Authors:** Alexandra Rucavado, Erika Camacho, Teresa Escalante, Bruno Lomonte, Julián Fernández, Daniela Solano, Isabel Quirós-Gutiérrez, Gabriel Ramírez-Vargas, Karol Vargas, Ivette Argüello, Alejandro Navarro, Carlos Abarca, Álvaro Segura, Jonathan Florentin, Hatem Kallel, Dabor Resiere, Remi Neviere, José María Gutiérrez

**Affiliations:** 1 Instituto Clodomiro Picado, Facultad de Microbiología, Universidad de Costa Rica, San José, Costa Rica; 2 Laboratorio de Hematología, Hospital Nacional de Niños ‘Dr Carlos Sáenz Herrera’, Caja Costarricense del Seguro Social, San José, Costa Rica; 3 Department of Toxicology and Critical Care Medicine, University Hospital of Martinique (CHU Martinique), Fort-de-France, France; 4 Intensive Care Unit, Cayenne General Hospital, Cayenne, French Guiana; 5 Tropical Biome and immunopathology CNRS UMR-9017, Inserm U 1019, Université de Guyane, Cayenne, French Guiana; 6 Cardiovascular Research Team (UR5_3 PC2E), University of the French West Indies (Université des Antilles), Fort de France, France; Universidade Nilton Lins, BRAZIL

## Abstract

**Background:**

The venom of *Bothrops lanceolatus*, a viperid species endemic to the Lesser Antillean Island of Martinique, induces thrombosis in a number of patients. Previous clinical observations indicate that thrombotic events are more common in patients bitten by juvenile specimens. There is a need to develop an experimental model of this effect in order to study the mechanisms involved.

**Methodology/Principal findings:**

The venoms of juvenile and adult specimens of *B*. *lanceolatus* were compared by (a) describing their proteome, (b) assessing their ability to induce thrombosis in a mouse model, and (c) evaluating their *in vitro* procoagulant activity and *in vivo* hemostasis alterations. Venom proteomes of juvenile and adult specimens were highly similar, albeit showing some differences. When injected by the intraperitoneal (i.p.) route, the venom of juvenile specimens induced the formation of abundant thrombi in the pulmonary vasculature, whereas this effect was less frequent in the case of adult venom. Thrombosis was not abrogated by the metalloproteinase inhibitor Batimastat. Both venoms showed a weak *in vitro* procoagulant effect on citrated mouse plasma and bovine fibrinogen. When administered intravenously (i.v.) venoms did not affect classical clotting tests (prothrombin time and activated partial thromboplastin time) but caused a partial drop in fibrinogen concentration. The venom of juvenile specimens induced partial alterations in some rotational thromboelastometry parameters after i.v. injection. When venoms were administered i.p., only minor alterations in classical clotting tests were observed with juvenile venom, and no changes occurred for either venom in rotational thromboelastometry parameters. Both juvenile and adult venoms induced a marked thrombocytopenia after i.p. injection.

**Conclusions/Significance:**

An experimental model of the thrombotic effect induced by *B*. *lanceolatus* venom was developed. This effect is more pronounced in the case of venom of juvenile specimens, despite the observation that juvenile and adult venom proteomes are similar. Adult and juvenile venoms do not induce a consumption coagulopathy characteristic of other *Bothrops* sp venoms. Both venoms induce a conspicuous thrombocytopenia. This experimental model reproduces the main clinical findings described in these envenomings and should be useful to understand the mechanisms of the thrombotic effect.

## Author summary

Envenomings by the viperid species *Bothrops lanceolatus*, endemic of the Caribbean Island of Martinique, are characterized by a thrombotic effect responsible for infarcts in various organs. Until now, no experimental *in vivo* models of this effect have been described. In this study, we developed a mouse model of thrombosis by using the intraperitoneal route of venom injection. The venom of juvenile specimens of *B*. *lanceolatus* induced the formation of abundant thrombi in the lungs, whereas the effect was much less pronounced with the venom of adult specimens. This difference in the ability of juvenile and adult venoms occurs despite both venoms having highly similar proteomic profiles. Both adult and juvenile venoms showed a weak *in vitro* procoagulant effect on plasma and fibrinogen, underscoring a thrombin-like (pseudo-procoagulant) activity. *In vivo*, the venoms did not affect the classical clotting tests (prothrombin time and activated partial thromboplastin time) but induced a partial drop in fibrinogen concentration and limited alterations in rotational thromboelastometry parameters when injected by the i.v. route. In contrast, few alterations of these parameters were observed after i.p. injection of venoms, in conditions in which thrombosis occurred, hence evidencing the lack of a consumption coagulopathy. After i.p. injection both venoms induced a pronounced thrombocytopenia. This experimental model reproduces some of the main clinical manifestations of envenoming by this species. This model can be used to identify the toxins responsible for the thrombotic effect, to study the mechanism(s) of thrombosis and to assess the preclinical efficacy of antivenoms and novel therapies.

## Introduction

*Bothrops lanceolatus* is a viperid snake species endemic to the Lesser Caribbean Island of Martinique as a result of a long-distance dispersal of South American species of the *Bothrops atrox-asper* complex [1]. It inflicts between 20–30 cases of envenoming per year [2,3]. The clinical manifestations of these envenomings include local effects, i.e., pain, edema, and necrosis, and systemic alterations, i.e., hemodynamic disturbances and, in some cases, alterations in hemostasis [2–5], most of which are characteristic of envenomings by *Bothrops* sp [6–8]. However, in contrast to the typical hemostatic alterations induced by *Bothrops* sp venoms, characterized by a consumption coagulopathy and defibrinogenation [8,9], some envenomings by *B*. *lanceolatus*, in the absence of antivenom treatment, involve a severe thrombotic effect which may result in cerebral, myocardial, and pulmonary infarctions [3,10,11], and a diffuse thrombotic microangiopathy [12]. Interestingly, clotting laboratory tests are altered to a much lower extent in these envenomings as compared to those inflicted by other *Bothrops* species, although thrombocytopenia is frequent [3,5,11]. This thrombotic effect has been also described in envenomings by the closely related species *B*. *caribbaeus*, endemic to the neighboring island of Saint Lucia [13].

Despite its clinical relevance, the pathogenic mechanisms and the toxins involved in this thrombotic effect remain unknown. It has been proposed that venom-induced alterations in the endothelium might be involved [12,13,14]. Other proposed mechanisms include platelet activation, the effect of venom on the binding of von Willebrand factor to type VI collagen in the subendothelium [15], and the proinflammatory activity of the venom [16–18].

Proteomic analysis of the venom of adult specimens of *B*. *lanceolatus* have revealed a pattern characteristic of viperid snake venoms, with predominance of P-III and P-I metalloproteinases (SVMPs), serine proteinases (SVSPs), phospholipases A_2_ (PLA_2_s) and, to a lower extent, L-amino acid oxidases and C-type lectin like proteins (SNACLECs), disintegrins, and cysteine-rich secretory proteins (CRISPs) [14,19]. The proteome and the toxicological profile of the venom of juvenile specimens have not been investigated. Experimental *in vitro* and *in vivo* studies with venom of adult specimens have documented lethal, hemorrhagic, edema-forming, myotoxic, PLA_2_, proteinase, fibrinogenolytic, complement-activating, and proinflammatory activities [14,16–18,20,21,22]. Conflicting results have been presented regarding the *in vitro* procoagulant activity of this venom on plasma, since this effect has been observed in some studies [19,23,24] but not in others [20,21]. On the other hand, the thrombotic effect described in humans has not been reproduced in mice injected intravenously (i.v.) with venom [14]. Thus, there is a need to develop experimental models which would allow the study of the mechanisms involved in this thrombotic effect.

It has been described that the thrombotic effect is more frequently observed in patients bitten by small, i.e., juvenile snakes [4,25], thus raising the possibility of ontogenetic variations in the composition and effects of the venom of this species. In order to develop an experimental model of venom-induced thrombosis, venoms were collected from juvenile and adult specimens to compare their proteomes and their effects on blood coagulation, platelet numbers, and thrombosis. Results revealed that the venom of juvenile specimens, when injected intraperitoneally, induces thrombosis in the pulmonary vasculature, whereas the venom of adult specimens has a much weaker thrombotic activity. This thrombotic effect occurs in the absence of a consumption coagulopathy characteristic of other *Bothrops* sp venoms.

## Methods

### Ethical statement

The methods carried out in this study using mice were approved by the Institutional Committee for the Care and Use of Laboratory Animals of Universidad de Costa Rica (approval code CICUA 13–2023). Mice (CD-1 strain, 20–23 g body weight of both sexes) were provided by the Bioterium of Instituto Clodomiro Picado. Mice were handled in Tecniplast Eurostandard Type II 1264C cages (268 x 215 x 141 mm), four mice per cage. Mice were maintained at 18–24°C, 60–65% relative humidity, and a 12:12 h light-dark cycle, and were provided with water and food *ad libitum*.

### Venoms

Venoms were obtained from *Bothrops lanceolatus* specimens caught in the wild in various locations of Martinique. Venom of adult snakes was a pool obtained from six specimens (five females, one male) with a range of body length of 150 to 172 cm. Venom of juvenile snakes was a pool prepared from ten specimens (five females, five males) with a range of body length of 59 to 92 cm. After venom extraction, snakes were released to the field in the places where they had been captured. Upon collection, venoms were frozen, freeze dried, and kept at -40 °C until used. Solutions of venoms were prepared immediately before use.

### SDS-Polyacrylamide gel electrophoresis (SDS-PAGE)

The venoms of *B*. *lanceolatus* (adults and juveniles) were comparatively analyzed by SDS-PAGE, after reduction with 2-mercaptoethanol for 5 min at 95°C. Samples of 10, 20 or 40 μg were loaded onto a 4–20% pre-cast gel (Bio-Rad; Hercules, CA, USA, catalog number 4561095) alongside with molecular weight markers (Bio-Rad, catalog number 1610375) and separated at 160 v in a mini-Protean apparatus (Bio-Rad, catalog number 1658000). Proteins were visualized with Coomassie Blue R-250 stain (Sigma-Aldrich, St. Louis, MO, USA, catalog number 42660) and recorded with ImageLab software (Version 6.1.0 build 7 Standard Edition).

### Reverse phase HPLC (RP-HPLC)

The venoms of *B*. *lanceolatus* (adults and juveniles) were comparatively analyzed by RP-HPLC. Samples of 2 mg were dissolved in 200 μL of water containing 0.1% trifluoroacetic acid (solution A) and centrifuged. The supernatant was applied to a reverse-phase column (C_18_, 250 x 4.6 mm, 5 μm particle; Phenomenex, Torrance, CA, USA, catalog number 00G-4053-E0) equilibrated with the same solution and separated at 1 mL/min using an Agilent 1220 chromatography system (Agilent Technologies, Santa Clara, CA, USA) monitored at 215 nm. Elution was carried out with a gradient toward acetonitrile containing 0.1% trifluoroacetic acid, as follows: 0% for 5 min, 0–15% for 10 min, 15–45% for 60 min, 45–70% for 10 min, and 70% for 9 min [26].

### Proteomic profiling

The venoms of *B*. *lanceolatus* (adults and juveniles) were comparatively analyzed using a bottom-up ’shotgun’ MS/MS approach, as described [27]. In brief, 15 μg of each venom, dissolved in 25 mM ammonium bicarbonate, were subjected to reduction with 10 mM dithiothreitol (30 min at 56 °C), alkylation with 50 mM iodoacetamide (20 min in the dark), and overnight digestion with sequencing grade trypsin at 37 °C. After stopping the reaction with 0.5 μL of formic acid, the tryptic peptides were dried in a vacuum centrifuge (Eppendorf, Hamburg, Germany, catalog number 022820168), redissolved in 0.1% formic acid, and separated by RP-HPLC on a nano-Easy 1200 chromatograph (Thermo Scientific, Waltham, MA, USA, catalog number LC140) coupled to a Q-Exactive Plus mass spectrometer (Thermo Scientific, catalog number IQLAAEGAAPFALGMBDK). Six μL of each sample (~0.7 μg of peptide mixture) were loaded onto a C18 trap column (75 μm × 2 cm, 3 μm particle; Thermo Scientific, catalog number 164943), washed with 0.1% formic acid (solution A), and separated at 200 nL/min on a C18 Easy-Spray PepMap column (75 μm × 15 cm, 3 μm particle; Thermo Scientific, catalog number ES900). A gradient toward solution B (80% acetonitrile, 0.1% formic acid) was developed for a total of 120 min (1–5% B in 1 min, 5–26% B in 84 min, 26–80% B in 30 min, 80–99% B in 1 min, and 99% B for 4 min). MS spectra were acquired in positive mode at 1.9 kV, with a capillary temperature of 200 °C, using 1 μscan in the range 400–1600 m/z, maximum injection time of 50 msec, AGC target of 1×10^6^, and resolution of 70,000. The top 10 ions with 2–5 positive charges were fragmented with AGC target of 3×10^6^, minimum AGC 2×10^3^, maximum injection time 110 msec, dynamic exclusion time 5 s, and resolution 17,500. MS/MS spectra were processed against protein sequences contained in the UniProt database for Serpentes (taxid:8570) using Peaks X (Bioinformatics Solutions, Waterloo, Canada, PEAKS Studio 10.0 build 20190129). Parent and fragment mass error tolerances were set at 15.0 ppm and 0.5 Da, respectively. Cysteine carbamidomethylation was set as fixed modification, while methionine oxidation and deamidation of asparagine or glutamine were set as variable modifications. A maximum of 2 missed cleavages by trypsin in semispecific mode were allowed. Filtration parameters for match acceptance were set to False Discovery Rate (FDR) <0.1%, detection of ≥1 unique peptide, and -10lgP protein score ≥30. A comparison of the number of matching/non-matching proteins of the SVMP and SVSP families between the juvenile and adult venoms was performed and represented by using Venn diagrams.

### *In vitro* coagulant activity on plasma and fibrinogen

Blood was collected from mice by cardiac puncture under inhaled isoflurane anesthesia, and immediately added to citrate anticoagulant (3.8% sodium citrate; citrate: blood volume ratio of 1:9), followed by centrifugation at 2,000 x g for 10 min for the collection of plasma. Aliquots of 200 μL of citrated plasma were incubated at 37°C for 5 min, and then 15 μL of 0.2 M CaCl_2_ was added, followed by 25 μL of various amounts of each venom, dissolved in 25 mM Tris-HCl, 137 mM NaCl, 3.4 mM KCl, pH 7.4 (TBS). Tubes were incubated at 37 °C and the clotting time recorded. The ability of venoms to clot fibrinogen was assessed by incubating 200 μL of a 6 mg/mL bovine fibrinogen solution (MP Biomedicals, Illkirch, France, catalog number 151122) at 37 °C for 5 min, followed by the addition of 25 μL of TBS containing various amounts of venoms. In both cases, the formation of a clot was visually assessed during a period of 10 min by tilting the tubes every minute. Procoagulant activity was also assessed by the turbidimetric assay described by O’Leary and Isbister [28], as modified by Sánchez et al. [29]. Briefly, 50 μg venom, dissolved in 100 μL TBS, were added to wells in a microplate and incubated for 2 min at 37°C in a microplate reader (Cytation 3 Imaging Reader, BioTek, VT, USA). Then, 4 μL of 0.4 M CaCl_2_ were added to 100 μL of mouse citrated plasma previously incubated at 37°C, and the mixture added to wells in the plate containing the venom dilutions. Controls included plasma/CaCl_2_ incubated with TBS in the absence of venom. After shaking for 5 sec, the absorbances at 340 nm were recorded during 10 min. In other experiments, the same protocol was followed, but a 6 mg/mL solution of bovine fibrinogen (Merck) was used instead of plasma, without the addition of CaCl_2_, in order to assess for thrombin-like (pseudo-procoagulant) activity. In this assay, various amounts of venom were tested and absorbances recorded at 10 min, and a dose of 25 μg venom was tested at various time intervals. In all cases, tests were done in triplicates.

### Effects of venom on coagulation parameters *in vivo*

Groups of four mice (20–22 g) received an i.v. injection of 20 μg of either juvenile or adult *B*. *lanceolatus* venom, dissolved in 100 μL 0.12 M NaCl, 0.04 M phosphate, pH 7.2 solution (PBS). One hr after injection, blood was collected by cardiac puncture under isoflurane anesthesia and immediately added to Eppendorf vials containing 3.8% sodium citrate as anticoagulant, using a citrate: blood volume ratio of 1: 9. In another set of experiments, a dose of 70 μg of either adult or juvenile *B*. *lanceolatus* venom, dissolved in 100 μL PBS, was injected by the intraperitoneal (i.p.) route into groups of four mice (20–22 g). Blood was collected 4 hr after injection and added to citrate anticoagulant, as described above. This dose was selected because it induced pulmonary thrombosis at this time interval (see below). For controls, groups of mice received an injection of 100 μL PBS by the i.v. route and were bled one hr after injection, as described.

Citrated blood samples were used for rotational thromboelastometry determinations, using a ROTEM Delta 4000 equipment according to the manufacturer’s instructions (Tem Innovations, GmbH, Munich, Germany). The parameters determined were: Extem, Intem and Fibtem clotting time (CT), clot formation time (CFT), and amplitude-clot strength at 20 min (A20). Extem and Intem tests evaluate the extrinsic and intrinsic coagulation pathways, respectively, whereas Fibtem evaluates the contribution of fibrinogen to clot formation and strength in conditions in which platelets are inhibited. CT is the time lapse (in sec) needed for the formation of a clot amplitude of 2 mm. CFT is the time lapse (in sec) between 2 mm clot amplitude and 20 mm clot amplitude. A20 reflects the clot firmness (in mm amplitude) 20 min after CT. These tests were run at a temperature of 37°C. Additional individual citrated blood samples from each experimental group were centrifuged at 2,000 g for 10 min, and plasma was obtained for determination of prothrombin time (PT) (STA-Neoplastine R, Stago, Paris, France), activated partial thromboplastin time (aPTT) (STA-PPT A, Stago) and fibrinogen concentration (STA-Liquid Fib, Stago), using a STA R Max2 coagulation analyzer (Stago). For platelet counts, a dose of 70 μg of either adult or juvenile *B*. *lanceolatus* venom, dissolved in 100 μL PBS, was injected i.p. into groups of four to eleven mice (20–22 g), whereas a control group of seven mice received 100 μL PBS alone. Blood was collected at 4 hr and added to sodium citrate as described. Platelet counts were carried out in citrated blood in an automated hematology analyzer (VetsCan HM5, Abaxis Global Diagnostics, Union City, CA, USA). The time of 4 hr was selected in the case of mice receiving an i.p. injection of venom because thrombi developed in the lungs at this time interval (see below).

### Histological assessment of thrombotic activity

In order to establish a model of the thrombotic activity described in patients envenomed by *B*. *lanceolatus*, various routes of venom injection were initially tested. Groups of four mice (22–23 g) received either 30 μg venom by the i.v. route in the caudal vein, 50 μg venom by the intramuscular (i.m.) route in the right gastrocnemius muscle, 50 μg by the subcutaneous (s.c.) route or 70 μg by the i.p. route, in all cases diluting the venom in 100 μL PBS. Groups of four control mice received 100 μL PBS under otherwise identical conditions. These venom doses were selected for being sublethal (in the case of i.v. and i.p. routes) on the basis of previous reports of Median Lethal Dose (LD_50_) of venom from adult specimens [21, 30], and for inducing prominent local tissue pathology without being lethal (in the cases of i.m. and s.c. routes). At either 4 or 24 hr after venom injection mice were sacrificed by cervical dislocation, and samples of heart, brain and lungs were obtained and added to 3.7% formalin fixative solution. Tissues were processed routinely and embedded in paraffin. Sections of 4 μm were obtained and stained with hematoxylin-eosin for microscopic observation.

### Staining for fibrin in the microvasculature

In order to ascertain whether fibrin microthrombi developed in the pulmonary microvasculature, paraffin-embedded sections from mice receiving i.p. injections of 70 μg of venom of juvenile specimens were prepared as described above and stained with the Martius-Scarlet-Blue kit for staining fibrin (Diapath, Martinengo, Italy, catalog number 010255). Sections of 4 μm were deparaffinized with xylene and ethanol and rehydrated with water. Then, sections were serially stained with martius yellow, crystal scarlet and methyl blue following the manufacturer’s instructions. Sections were briefly rinsed with 1% acetic acid, dehydrated and mounted. With this method, fibrin stains red, erythrocytes yellow, and connective tissue blue.

### Role of SVMPs in the thrombotic effect

Once the experimental model of thrombotic effect was established, the role of SVMPs in the pathogenesis of this effect was assessed by using the metalloproteinase inhibitor Batimastat (British Biotech, Oxford, UK). Solutions of venom from adult or juvenile specimens were prepared in PBS and incubated for 30 min at room temperature with Batimastat (final concentration 250 μM). This concentration was selected to ensure complete inhibition of SVMPs on the basis of a previous study with venom of a closely related species showing that proteolytic and hemorrhagic activities were inhibited at concentrations lower than 250 μM of the inhibitor when incubated prior to injection [31]. Solutions of venoms incubated with the vehicle alone were also prepared. Aliquots of 100 μl of the mixtures, containing 70 μg venom, were injected i.p. into groups of four mice (22–23 g). Four hr after injection, mice were sacrificed and tissue samples from the lungs were obtained and processed for histological observation, as described above.

### Statistical analyses

Results were expressed as mean ± SEM. The significance of the differences between the mean values of experimental groups was assessed by Mann-Whitney U test when two groups were compared. In experiments involving more than two groups the significance of the differences was assessed by one-way ANOVA for normally distributed data and by Kruskal-Wallis test for non-normally distributed data. Tukey-Kramer or Dunn’s post-hoc tests, respectively, were used to analyze differences between pairs of mean values. Other results with non-normally distributed data were analyzed by Generalized Linear Model (GLM), which was initially tried using a binomial or poisson distribution but was changed to a quasibinomial or quasipoisson distribution due to overdispersion. P values < 0.05 were considered significant.

## Results

### Electrophoretic and chromatographic analyses of the venoms

**Fig 1:**
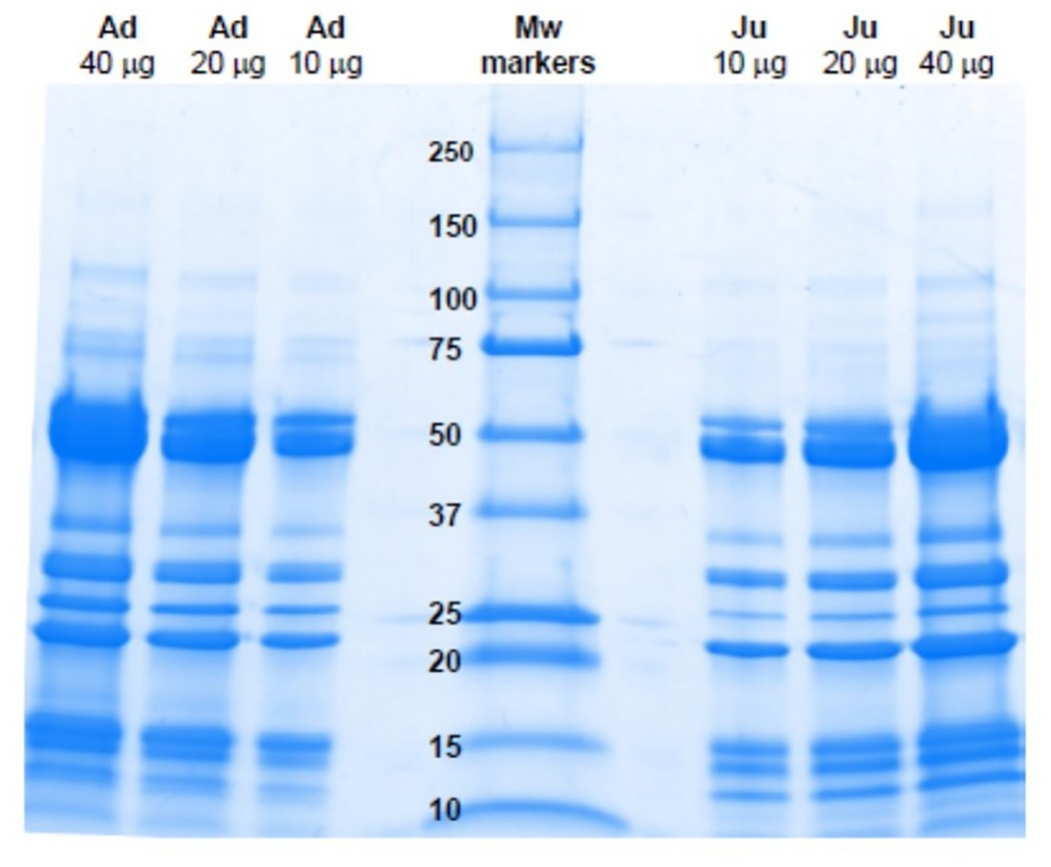
SDS-PAGE separation of venoms of adult (Ad) and juvenile (Ju) specimens of *B*. *lanceolatus*. Various amounts of venom (10, 20 and 40 μg) were separated under reducing conditions on 4–20% pre-cast gels. Molecular weight (Mw) markers were also run. Proteins were stained with Coomassie Blue R-250.

**Fig 2:**
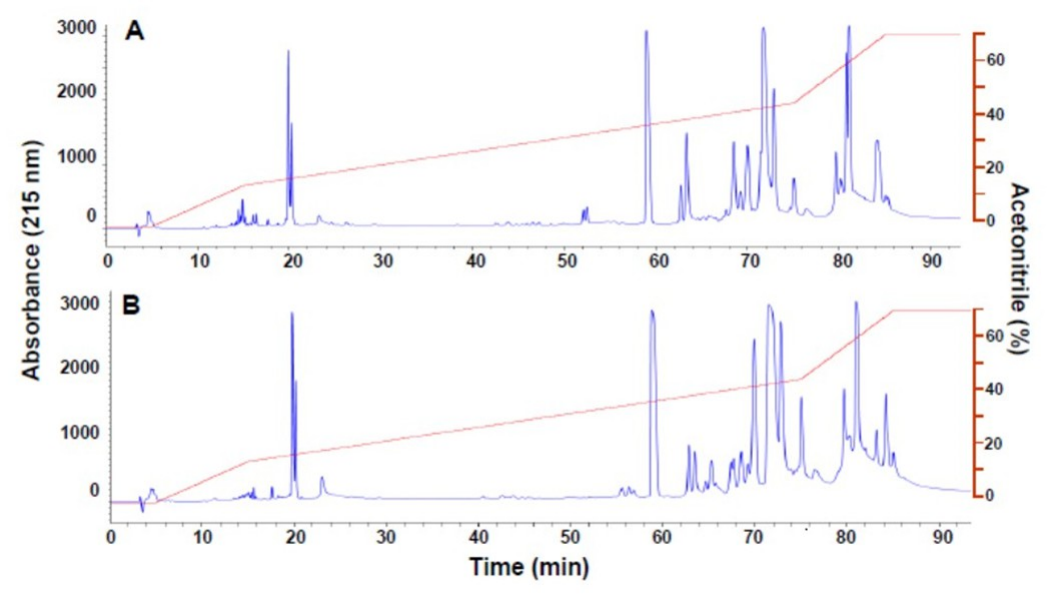
RP-HPLC separation of venoms of juvenile (A) and adult (B) specimens of *B*. *lanceolatus*. Samples of 2 mg were dissolved in water containing 0.1% trifluoroacetic acid (solution A). After centrifugation, the supernatant was applied to a reverse-phase column equilibrated with solution A and separation was monitored by recording the absorbance at 215 nm. The following gradient of acetonitrile, containing 0.1% trifluoroacetic acid, was used for elution: 0% for 5 min, 0–15% for 10 min, 15–45% for 60 min, 45–70% for 10 min, and 70% for 9 min (red line).

SDS-PAGE separation of venoms revealed highly similar electrophoretic patterns (Fig 1), with bands in the range of 150 kDa to 10 kDa. The most abundant bands have estimated molecular masses of 50, 30, 25, 22 and 15 kDa. A qualitatively similar RP-HPLC profile was also observed in these venoms (Fig 2A and 2B), with some differences in peaks eluting at 52 min (juvenile venom) and 58, 65 and 83 min (adult venom), and in the region between 60 and 70 min.

### Proteomic profiling

Table 1 depicts the protein families identified in the venoms of *B*. *lanceolatus* (adults and juveniles) by bottom-up shotgun proteomics. Families present in both venoms include metalloproteinases (SVMPs), serine proteinases (SVSPs), phospholipases A_2_ (PLA_2_), C-type lectin-like proteins, L-amino acid oxidases, nerve growth factor, phospholipase B, and glutamyl cyclase, with highest number of variants in the first four families (Table 1). On the other hand, nucleotidase, vascular endothelial growth factor, hyaluronidase and PLA_2_ inhibitor were detected only in the adult venom, whereas protein disulfide isomerase was detected only in juvenile venom (Table 1). S1 Table provides the details of the protein matches and supporting peptides. A comparison of the number of matching proteins of the SVMP and SVSP families between the juvenile and adult venoms was performed. As shown in the Venn diagrams (S1 Fig) proteins identified as SVSP showed a higher proportion of shared matches, in comparison to proteins of the SVMP family.

**Table 1:** Protein families identified in the venoms of *B*. *lanceolatus* (adults and juveniles) by bottom-up shotgun proteomics*.

Protein family	Juveniles	Number of variants**	Adults	Number of variants
Metalloproteinase	**√**	22	**√**	25
Serine proteinase	**√**	22	**√**	21
Phospholipase A_2_	**√**	9	**√**	13
C-type lectin/lectin-like	**√**	13	**√**	12
L-amino acid oxidase	**√**	2	**√**	2
Phosphodiesterase	**√**	2	**√**	2
Nerve growth factor	**√**	1	**√**	1
Natriuretic peptide/BPP	**√**	2	(-)	(-)
Phospholipase B	**√**	1	**√**	1
Glutaminyl cyclase	**√**	1	**√**	1
Protein disulfide-isomerase	**√**	1	(-)	(-)
Nucleotidase	(-)	(-)	**√**	1
Vascular endothelial growth factor	(-)	(-)	**√**	2
Hyaluronidase	(-)	(-)	**√**	1
Phospholipase A_2_ inhibitor	(-)	(-)	**√**	1
**Total number**	**11**	**76**	**14**	**83**

* For a detailed summary of protein matches and supporting peptides, refer to S1 Table.

** Number of variants is expressed as the minimum number of distinct ‘protein groups’ calculated by the Peaks X software, supported by at least 1 unique peptide.

√: detected; (-) not detected.

### *In vitro* procoagulant activity on plasma and fibrinogen

**Fig 3:**
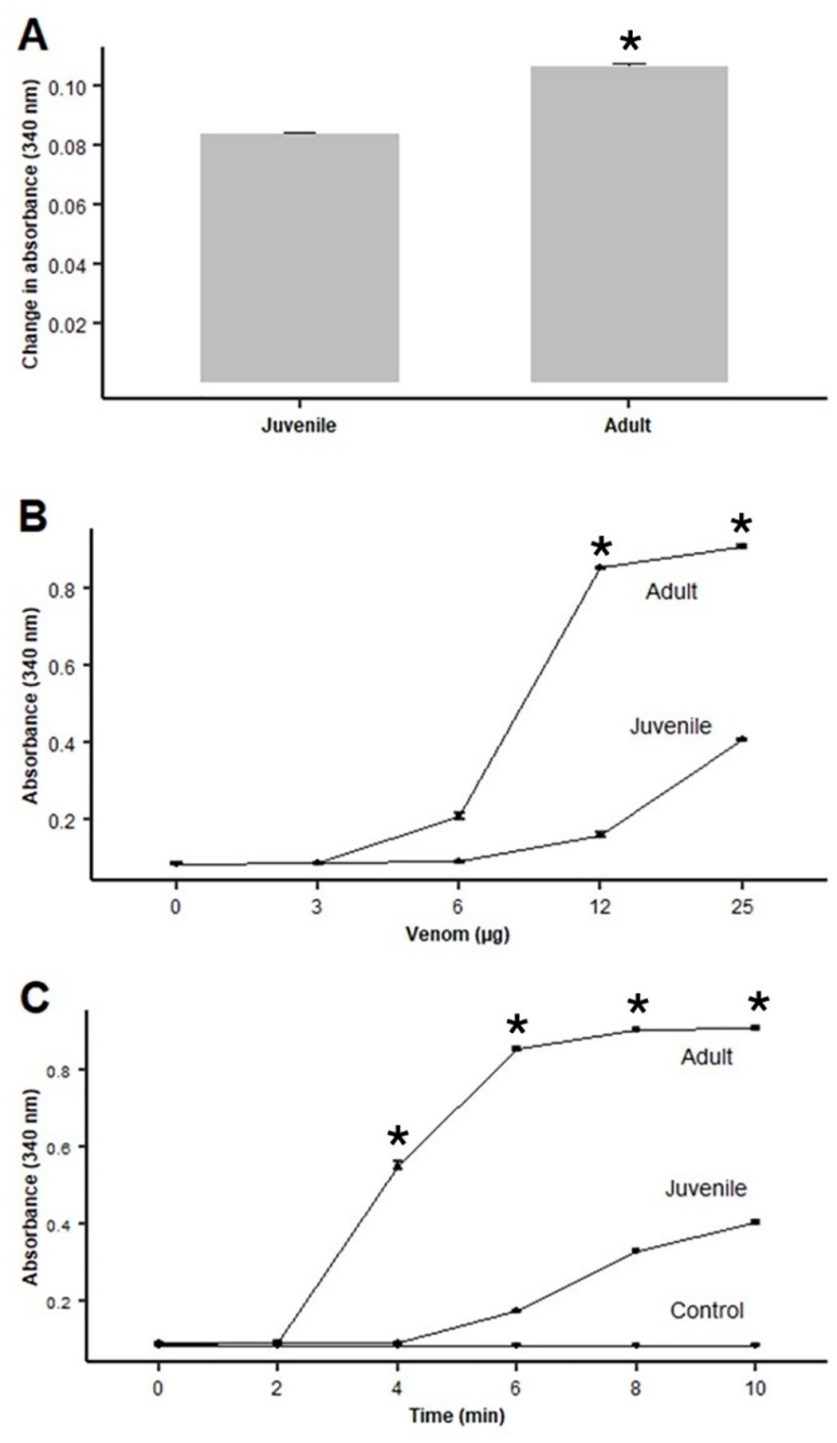
*In vitro* procoagulant activity of venoms of juvenile and adult specimens of *B*. *lanceolatus*. A: Procoagulant effect on plasma. Fifty μg venom, dissolved in 100 μL TBS, were added to wells in a microplate. Then, 4 μL of 0.4 M CaCl_2_ were added to 100 μL of mouse citrated plasma previously incubated at 37°C, and the mixture added to wells in the plate containing the venom solution. Controls included plasma/CaCl_2_ incubated with TBS with no venom. After shaking for 5 sec, the absorbances at 340 nm were recorded during 10 min as an index of the increase in turbidity of the samples. No change in absorbance was observed in control samples. B and C: Thrombin-like (pseudo-procoagulant) activity effect of venoms on bovine fibrinogen. In B, solutions containing various amounts of venom, dissolved in 100 μL TBS, were added to 100 μL of a fibrinogen solution (6 mg/mL) previously incubated at 37°C, and the change in absorbance at 340 nm were recorded at 10 min. In C, solutions containing 25 μg venom, dissolved in 100 μL TBS, were added to 100 μL of fibrinogen (6 mg/mL). The changes in absorbance at 340 nm were recorded at various time intervals. Controls included fibrinogen incubated with TBS with no venom. No change in absorbance was observed in control samples. Results are presented as mean ± SEM (n = 3). *p < 0.05.

When added to citrated plasma in the presence of calcium, the venom of juvenile specimens of *B*. *lanceolatus* did not induce the formation of a firm clot up to a dose of 50 μg for a maximum observation period of 10 min, although an increase in turbidity was observed. When testing the venom of adult specimens, a weak clot formed after approximately 10 min of incubation when using a dose of 50 μg venom, whereas no clot formation, and only an increase in turbidity, occurred when using doses of venom of 25 μg, 12.5 μg, and 6.25 μg. When venoms were added to a bovine fibrinogen solution, the venom of adult specimens induced the formation of a fibrin clot in a dose-dependent way. The time required to form a visible clot was 10 min, 9 min, 4.5 min and 3 min for venom amounts of 6.25 μg, 12.5 μg, 25 μg and 50 μg, respectively. In contrast, the venom of juvenile specimens did not induce the formation of a firm fibrin clot when the same doses were tested, although an increase in turbidity was observed at the dose of 50 μg. When the more sensitive turbidimetric method was used, both venoms induced an increase in absorbance at 340 nm, reflecting the formation of fibrin after addition of venoms to plasma (Fig 3A) and fibrinogen (Fig 3B and 3C), thus revealing a thrombin-like (pseudo-procoagulant) activity. Venom of adult specimens showed a stronger thrombin-like activity than venom of juvenile specimens.

### Effects of venoms on coagulation parameters *in vivo*

**Fig 4:**
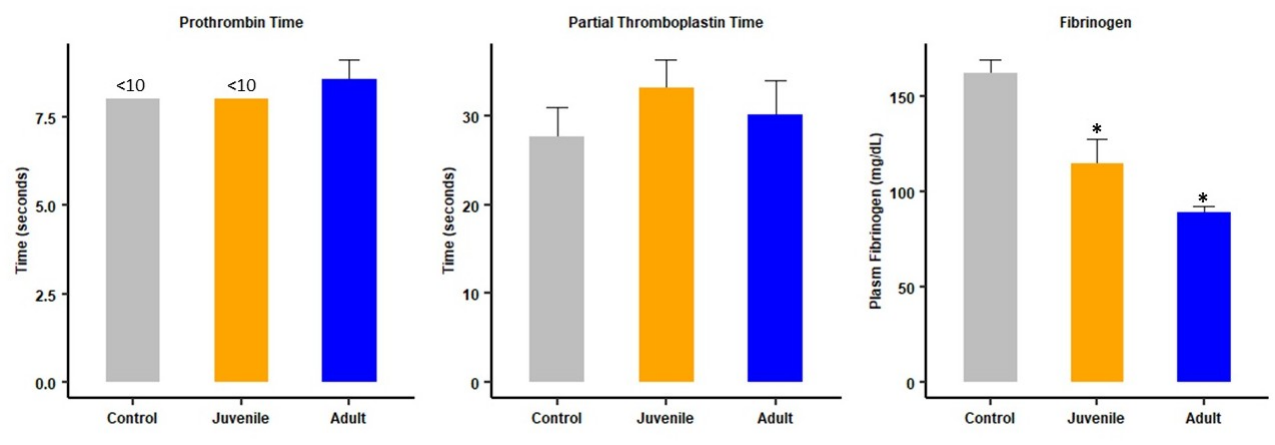
Effect of i.v. injection of juvenile and adult *B*. *lanceolatus* venoms on classical clotting tests. 20 μg venom from juvenile or adult specimens, dissolved in 100 μL PBS, were injected i.v. in mice. Controls received 100 μl PBS. One hour after injection mice were bled under isoflurane anesthesia and blood was collected, added to citrate anticoagulant, and centrifuged for plasma collection to determine prothrombin time (PT), activated partial thromboplastin time (aPTT) and fibrinogen concentration. Results are presented as mean ± SEM (n = 4). *p < 0.01 when compared to controls.

Experiments were done in order to assess the effect of venoms of juvenile and adult specimens on classical clotting tests and rotational thromboelastometry parameters. For this, two experimental settings were used: i.v. injection of 20 μg venom followed by blood collection and testing one hr after injection, and i.p. injection of 70 μg venom followed by blood collection and testing at 4 hr, the time when thrombosis was observed in pulmonary blood vessels. When the i.v. route was used, no major changes were observed in PT and aPTT in envenomed mice receiving either venom, as compared to control mice receiving PBS. However, a partial and significant drop in fibrinogen concentration occurred at one hr after i.v. injection of both venoms (Fig 4). No significant differences (p > 0.05) were observed when comparing the effect of the two venoms on fibrinogen concentration.

**Fig 5:**
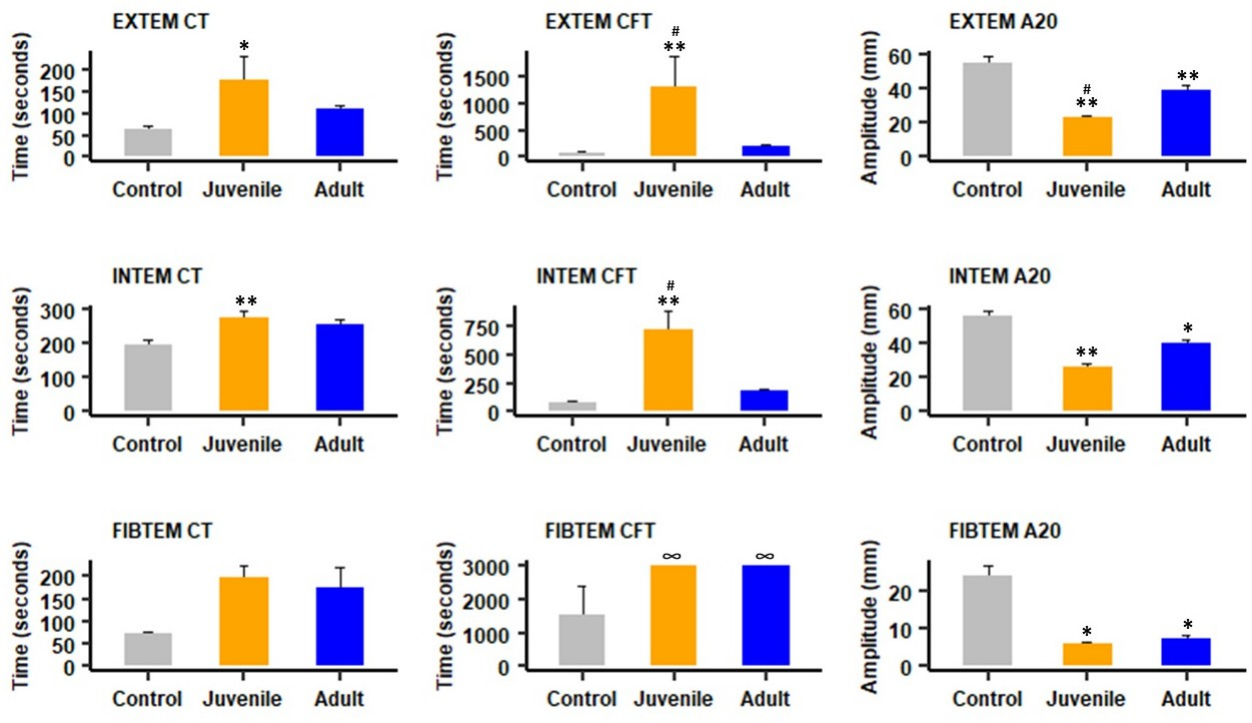
Effect of i.v. injection of juvenile and adult *B*. *lanceolatus* venoms on rotational thromboelastometry parameters. Twenty μg venom, dissolved in 100 μL PBS, was injected i.v. in mice. Controls received 100 μl of PBS. On hour after injection mice were bled under isoflurane anesthesia and blood was collected and added to citrate anticoagulant for determination of Extem, Intem and Fibtem parameters. CT: Clotting time; CFT: clot formation time; A20: clot amplitude (in mm) 20 min after CT (see Methods for details). Results are presented as mean ± SEM (n = 4). *p < 0.05, **p<0.01 when compared to control; # p < 0.05 when comparing juvenile and adult venoms. ∞: In the case of Fibtem CFT in envenomed mice, the clot did not reach an amplitude of 20 mm.

**Fig 6:**
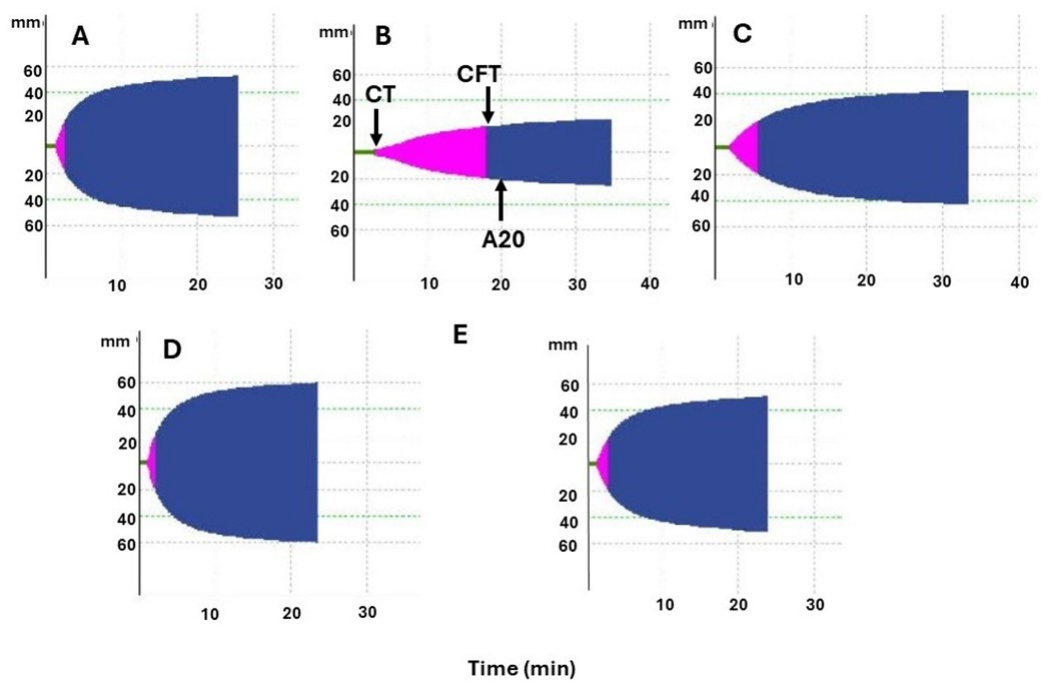
Representative Extem rotational thromboelastometry tracings from mice injected with PBS or venoms of juvenile and adult specimens of *B*. *lanceolatus*. Blood was collected by cardiac puncture under inhaled isoflurane anesthesia, added to sodium citrate solution, and evaluated by rotational thromboelastometry (see Methods for details). (A) Extem tracing from a mouse receiving PBS by the i.v. route and bled 1 hr after injection. (B) and (C) Extem tracings from mice receiving 20 μg of juvenile (B) or adult (C) *B*. *lanceolatus* venoms by the i.v. route and bled 1 hr after injection. (D) and (E) Extem tracings from mice receiving 70 μg of juvenile (D) or adult (E) venoms by the i.p. route and bled 4 hr after injection. CT (clotting time, green line) is the time lapse (in sec) needed for the formation of a clot amplitude of 2 mm. CFT (clot formation time, pink) is the time lapse (in sec) between 2 mm clot amplitude and 20 mm clot amplitude. A20 (clot amplitude at 20 min) reflects the clot firmness (in mm amplitude) 20 min after CT.

Regarding rotational thromboelastometry parameters in samples obtained 1 hr after i.v. injection, Extem and Intem CT and CFT, and Fibtem CFT were prolonged in mice injected with juvenile venom, as compared to controls, whereas only Fibtem CFT was prolonged in the case of adult venom. When the clot strength was assessed by the determination of the A20 parameter, it was altered in Extem, Intem and Fibtem in the case of juvenile and adult venoms (Figs 5 and 6). When comparing parameters in juvenile and adult venoms, significant differences were observed in Extem CFT, Intem CFT and Extem A20, juvenile venom showing higher alterations than adult venom (Fig 5). Thus, overall, the venom of juvenile specimens induced more pronounced alterations in these parameters than the venom of adult specimens when injected by the i.v. route. S2 and S3 Figs depict Intem and Fibtem rotational thromboelastometry tracings.

**Fig 7:**
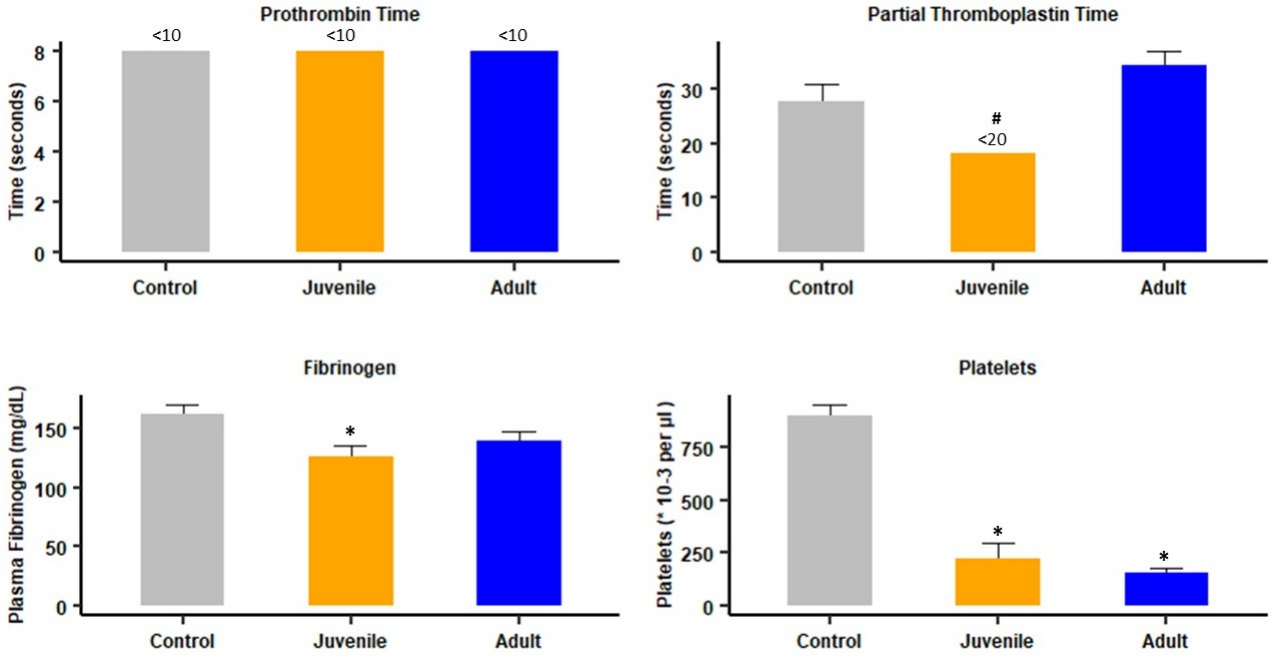
Effects of i.p. injection of juvenile and adult *B*. *lanceolatus* venoms on classical clotting tests and platelet counts. Mice received 70 μg venom, dissolved in 100 μL PBS, by the i.p. route. Controls received 100 μl of PBS. Four hours after injection mice were bled under isoflurane anesthesia and blood was collected and added to citrate anticoagulant for determination of prothrombin time (PT), activated partial thromboplastin time (aPTT), fibrinogen concentration, and platelet counts (see Methods for details). Results are presented as mean ± SEM (n = 4 in the case of clotting tests and fibrinogen concentration and n = 4–11 in the case of platelet counts). *p < 0.05 when compared to control; # p < 0.05 when comparing juvenile and adult venom.

In order to assess the status of clotting parameters in circumstances when pulmonary thrombosis occurred, the same assays were carried out in samples collected 4 hr after i.p. injection of 70 μg venom, which induces thrombosis. No alterations in PT, aPTT and fibrinogen concentration were observed in mice receiving adult venom, whereas a partial, but significant, change occurred in aPTT and fibrinogen concentration in mice receiving juvenile venom (Fig 7). On the other hand, both venoms induced a profound thrombocytopenia, as compared to controls (Fig 7). No significant differences (p > 0.05) were observed when comparing the effect of the two venoms on platelet counts.

**Fig 8:**
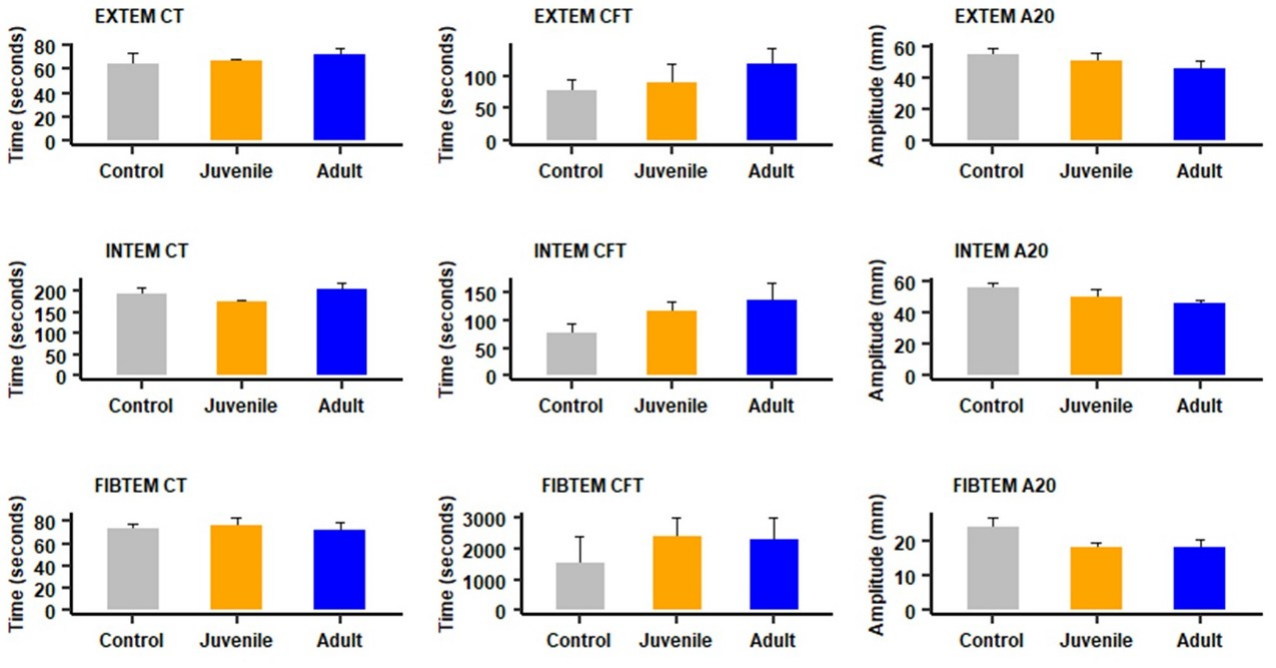
Effect of i.p. injection of juvenile and adult *B*. *lanceolatus* venoms on rotational thromboelastometry parameters. Mice received 70 μg of venom, dissolved in PBS, by the i.p. route. Controls were injected with 100 μl of PBS. Four hours after injection mice were bled under isoflurane anesthesia and blood was added to citrate anticoagulant for determination of Extem, Intem and Fibtem parameters. CT: Clotting time; CFT: clot formation time; A20: clot amplitude (in mm) 20 min after CT (see Methods for details). Results are presented as mean ± SEM (n = 4). No significant differences (p > 0.05) were observed between the groups.

Analysis of rotational thromboelastometry parameters in blood samples collected 4 hr after i.p. injection of venoms showed no significant alterations in samples from envenomed mice, as compared to those from control mice (Figs 6, 8, S2, and S3). The raw data related to coagulation, rotational thromboelastometry and platelet counts are available in S2 Table.

### Histological assessment of thrombotic activity

**Fig 9:**
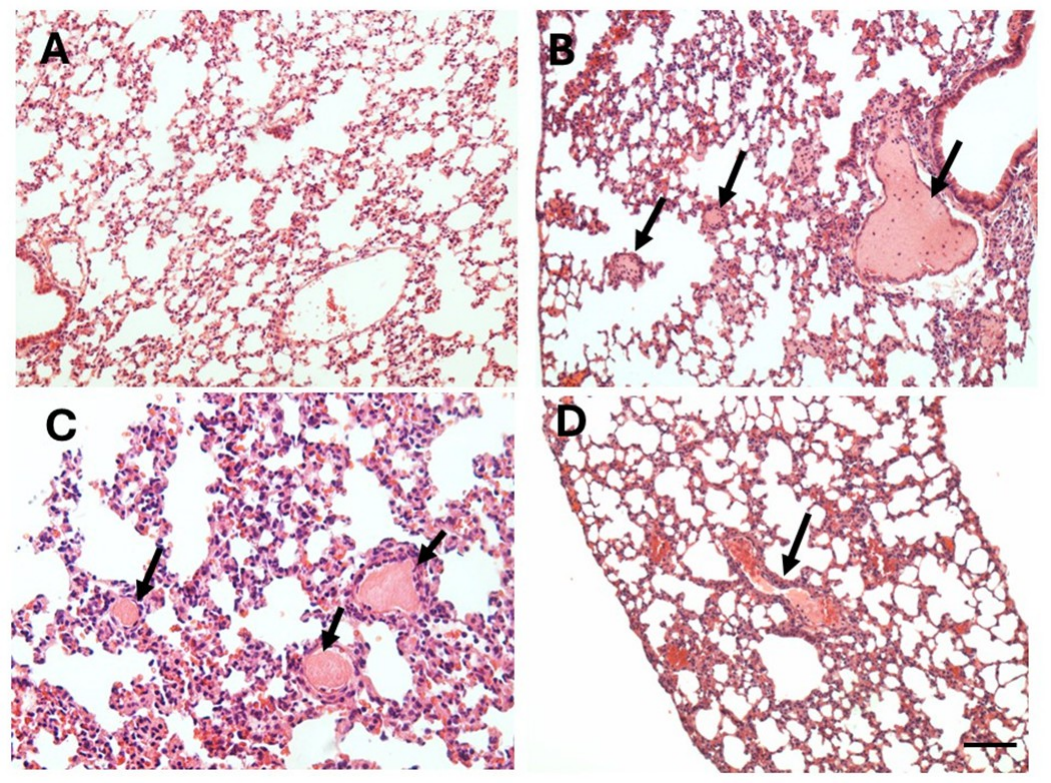
Light micrographs of sections of pulmonary tissue of mice which received an i.p. injection of venom from juvenile or adult *B*. *lanceolatus*. Sections correspond to samples of mice receiving 100 μL PBS (A) or 70 μg of *B*. *lanceolatus* venom from either juvenile (B and C) or adult (D) specimens, dissolved in 100 μL PBS. Mice were sacrificed 4 hr after injections, and samples from pulmonary tissue were collected, added to formalin fixative and processed for embedding in paraffin. Sections from control mice receiving PBS show a normal histological pattern. Abundant thrombi are observed in sections of mice receiving juvenile venom (arrows in B and C). In contrast, samples from mice injected with adult venom are largely devoid of thrombi, and only a pale hyaline material is observed in some vessels (arrow in D). Hematoxylin-eosin staining. Bar represents 100 μm.

Tissue samples from control mice receiving injections of PBS by various routes showed normal histological features in heart, brain, and lungs. In the case of lungs, the normal histological patterns of bronchi, bronchioles, alveolar sacs and blood vessels was preserved (Fig 9A). In the cases of animals receiving venoms, no thrombi were observed in brain, heart, and lung blood vessels after adult and juvenile *B*. *lanceolatus* venom injections by the i.v., s.c. and i.m. routes at 4 hr and 24 hr. In contrast, when 70 μg venom were administered by the i.p. route, the venom of juvenile *B*. *lanceolatus* specimens induced numerous thrombi in the lungs at both time intervals (Fig 9B and 9C), but not in heart or brain. Few mice receiving 70 μg juvenile venom by the i.p. route died before 4 hr; in these cases, additional mice were injected to complete the sample of four mice per time interval. In samples from mice receiving juvenile venom by the i.p. route thrombi were observed in vessels of variable caliber, being more abundant in veins, although they were also present in arteries and arterioles. On the other hand, venom of adult specimens induced the formation of thrombi only in few blood vessels and in few of the tissue samples analyzed, and no thrombi were observed in brain and heart vasculature. In some pulmonary vessels, mostly veins, of mice injected with adult venom a hyaline pale material was observed having a lower density as compared to the overt thrombi observed with juvenile venom (Fig 9D). No mice injected with adult venom by the i.p. route died. Venoms of both juvenile and adult specimens induced hemorrhage in the lungs, evidenced by the presence of erythrocytes in the alveolar spaces in some regions.

Overall, blood vessels with thrombi were more abundant in mice injected with juvenile venom than in those receiving adult venom. In order to provide a semiquantitative assessment of the frequency of thrombi formation, a number of histological sections obtained from mice injected with venoms were examined. Among 26 sections from mice receiving juvenile venom, 20 showed thrombi, 3 presented the hyaline pattern of staining described above, and 3 did not show thrombi. In contrast, in the case of 16 sections from lung tissue of mice injected with adult venom, 1 had thrombi, 6 presented the hyaline material inside vessels, and 9 did not show thrombi or hyaline material.

### Staining for fibrin in the microvasculature

**Fig 10:**
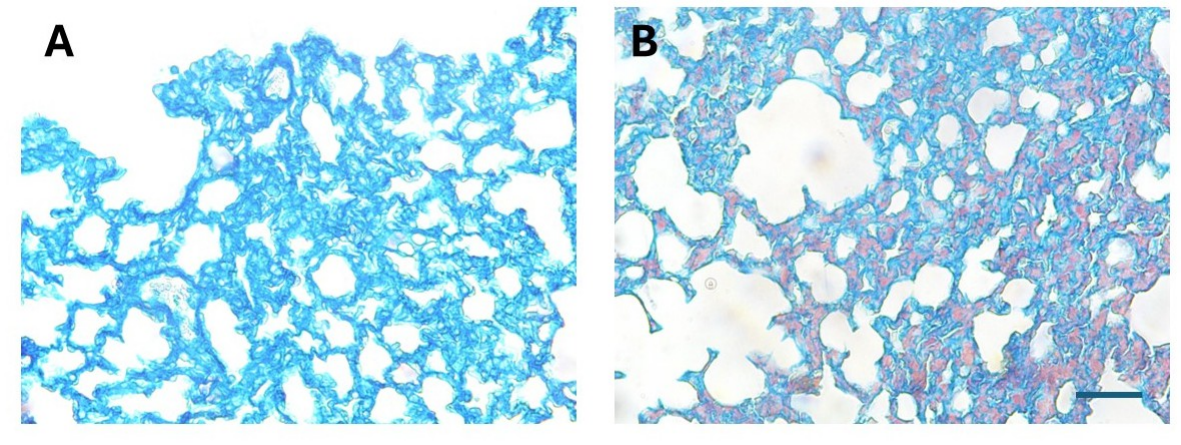
Thrombi in the pulmonary microvasculature of mice injected with juvenile *B*. *lanceolatus* venom. Light micrographs of sections of pulmonary tissue of mice which received an i.p. injection of 100 μL PBS (A) or 70 μg of *B*. *lanceolatus* venom from juvenile specimens, dissolved in 100 μL PBS (B). Mice were sacrificed 4 hr after injections, and samples from pulmonary tissue were collected, added to formalin fixative and processed for embedding in paraffin. Sections were stained with Martius-Scarlet-Blue, which stains fibrin in red color. No fibrin is observed in samples of mice receiving PBS, whereas abundant red fibrin deposits are observed in the microvasculature of the section from mice treated with venom. Bar represents 100 μm.

It was of interest to assess whether thrombi were also present in the microvasculature of mice receiving juvenile venom by using a specific staining of fibrin (Martius-Scarlet-Blue). No red staining, characteristic of fibrin, was observed in the microvasculature of alveolar septa in samples from mice injected with PBS (Fig 10A), whereas abundant red-stained microthrombi occurred in sections from mice that had received an i.p. injection of venom from juvenile specimens 4 hr after injection (Fig 10B).

### Role of SVMPs in the thrombotic effect

**Fig 11:**
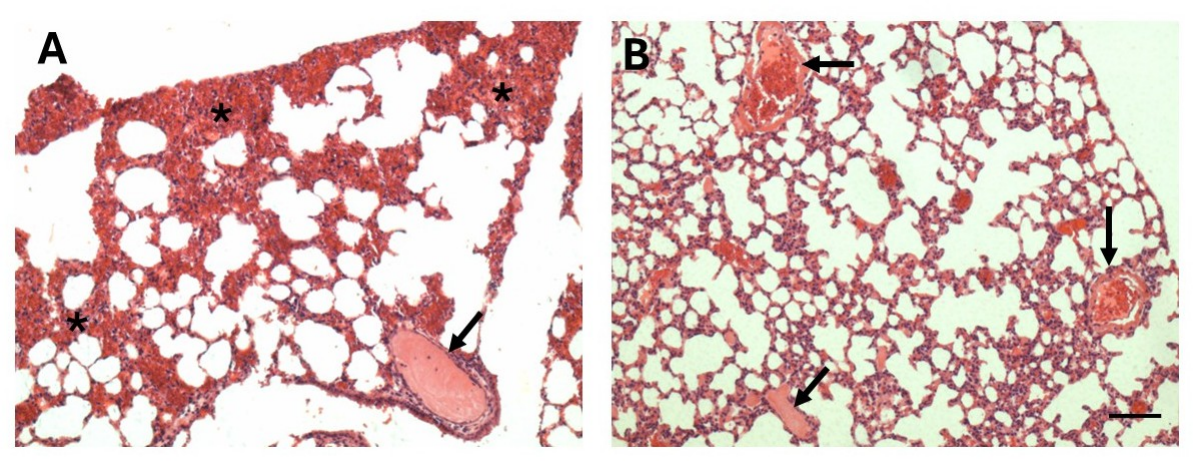
Inhibitory action of Batimastat on the effects induced by juvenile *B*. *lanceolatus* venom in the lungs. Light micrographs of sections of pulmonary tissue of mice which received an i.p. injection of 70 μg of *B*. *lanceolatus* venom from juvenile specimens dissolved in 100 μL PBS (A) or the same dose of venom which was previously incubated with Batimastat (250 μM final concentration) (B). Mice were sacrificed 4 hr after injections, and samples from pulmonary tissue were collected, added to formalin fixative and processed for embedding in paraffin. The section from a mouse receiving venom show abundant hemorrhage (*) and a thrombus in a blood vessel (arrow). In contrast, no hemorrhage is observed in tissue from a mouse receiving venom incubated with Batimastat, whereas thrombi are present (arrows). Hematoxylin-eosin staining. Bar represents 100 μm.

In order to assess the role of SVMPs in the formation of pulmonary thrombi, venoms were incubated with the metalloproteinase inhibitor Batimastat before i.p. injection. Samples from mice injected with venom incubated with PBS showed thrombi in blood vessels and hemorrhage, evidenced by abundant erythrocytes in the alveolar spaces (Fig 11A). Incubation of venom with Batimastat did not inhibit the formation of thrombi in samples injected with juvenile venom (Fig 11B), and also did not prevent the formation of the intravascular hyaline material in the case of adult venoms. In contrast, Batimastat was effective in the inhibition of hemorrhagic effect in the lungs (Fig 11B). Thus, SVMPs do not seem to be the causative agent of pulmonary thrombosis in this model but are responsible for the hemorrhagic activity. The raw data related to histological assessment of thrombosis are available in S3 Table.

## Discussion

The mouse experimental model described in this work reproduces three of the characteristic clinical findings in patients envenomed by *B*. *lanceolatus*, i.e., thrombosis, lack of consumption coagulopathy, and thrombocytopenia. Clinical observations indicate that patients developing thrombosis were generally bitten by specimens of small size [4, 22]. This prompted us to obtain venom of juvenile snakes and compare its composition and effects with those of adult specimens. Results show that, when administered by the i.p. route, venom from juvenile specimens induced pulmonary thrombosis in mice, whereas the venom of adult specimens induced this effect to a much lower extent. Interestingly, no such effect was observed when venom was injected i.v., s.c. or i.m. When administered by the i.p. route, macromolecules reach the systemic circulation mainly via lymphatic vessels and reach higher concentrations in blood than when using the s.c. and i.m. routes [32]. It is suggested that the i.p. route is a model of systemic absorption of the venom that, in terms of toxicokinetics, may resemble what occurs in clinical cases. Thus, our model could be used to study the mechanism of thrombosis in *B*. *lanceolatus* envenomings.

The majority of thrombi observed in pulmonary vasculature in mice injected with juvenile venoms were observed in large and medium size veins, although thrombi were also present in arteries and arterioles. In addition, a staining for fibrin showed positive staining in the microvasculature of the alveolar septa, hence reproducing the phenomenon of diffuse thrombotic microangiopathy described in an autopsy of a patient envenomed by *B*. *lanceolatus* [12]. In our study we did not observe thrombi in cerebral or myocardial blood vessels, which are common findings in human patients [2–4]. Our observations can be explained by the higher propensity of the pulmonary vasculature to develop inflammatory and thrombotic complications in a variety of diseases [33,34] and by its unique hemodynamic and immunologic features. In the case of tissue samples from mice injected with adult venoms the occurrence of evident thrombi was infrequent. Instead, a pale hyaline material was observed in some vessels. Clearly, the thrombotic action induced by adult venoms was weaker than by juvenile venoms.

The proteomic analysis of juvenile and adult venoms did not reveal overt differences in composition. Thus, *B*. *lanceolatus* presents a venom proteomic profile that fits within the ‘paedomorphic’ pattern described for some populations of *B*. *atrox*, in which there are no major changes in venom composition as snakes age [35]. *Bothrops* sp venoms present a dichotomic ontogenetic pattern, with some venoms having a ‘paedomorphic’ profile while others show drastic changes as snakes age, corresponding to an ‘ontogenetic’ profile [35,36]. Thus, the basis for the difference in thrombogenic potential between juvenile and adult venoms is not evident from the overall electrophoretic, chromatographic, and proteomic comparison. However, when the matches of protein sequences were analyzed, it was found that SVMPs showed a lower proportion of shared matches than in the case of SVSPs. This suggests that, despite the overall similarities in these venoms, variations in the isoforms present in toxins within the most abundant protein families may account for the functional difference observed, an issue that awaits the identification of the thrombogenic factor(s) in venoms of juvenile specimens. The small amount of venom available from juvenile specimens did not allow us to undertake the purification of venom components.

The use of the SVMP inhibitor Batimastat allowed us to assess whether SVMPs are involved in the thrombotic effect. It has been hypothesized that *B*. *lanceolatus* venom may activate the vascular endothelium, rendering it thrombogenic [12,37], an effect that might be related to the action of SVMPs, which are abundant in this venom [14,19,38,39]. It is known that SVMPs induce a variety of effects on endothelial cells [40–42]. However, inhibition of SVMPs did not prevent the formation of thrombi, although abrogated pulmonary hemorrhage, thus evidencing that thrombosis occurs in conditions where SVMPs are inhibited and implying that other as yet unidentified components are responsible for this effect. The proinflammatory effect of *B*. *lanceolatus* venom, reflected by its ability to activate the complement system and generate a variety of mediators, has been proposed as a possible mechanism of the thrombotic effect [16–18].

Alternative mechanisms of thrombosis might be related to the action of venom on von Willebrand factor, promoting its binding to type VI collagen in the subendothelial surface [15] or to platelet activation, perhaps associated with the thrombocytopenia observed in clinical cases and in our experimental conditions. Additionally, the release of tissue factor, as a consequence of the action of venom in the vasculature, might be involved in the thrombotic effect. Likewise, the possible role of SVSPs in the genesis of thrombosis, either by acting on clotting factors or on platelets should be explored in future studies. It is likely that the pathogenesis of thrombosis involves an interplay between a variety of mechanisms, including inflammatory events and platelet alterations in a scenario of thromboinflammation [43,44]. There might also be underlying factors related to the conditions of the patients which could contribute to thrombogenesis. On the other hand, our observations concur with clinical laboratory findings in that this venom induces thrombocytopenia, which might be a consequence of the action of a C-type lectin-like component, similar to the one characterized from the closely related venom of *B*. *caribbaeus* [45].

There have been conflicting findings in the literature concerning the effect of *B*. *lanceolatus* venom on hemostasis *in vitro* and *in vivo*. Regarding the *in vitro* procoagulant effect on citrated plasma, both negative and positive results have been described [14,19,21,23,24], whereas the venom did not induce defibrinogenation in mice [21]. It has been proposed that the negative *in vitro* results described by Bogarín et al. [21] and Gutiérrez et al. [14] are due to the fact that calcium, a cofactor required for coagulation, was not added to the plasma in these experiments. When calcium is added, adult *B lanceolatus* venom induces plasma clotting *in vitro* [19,23,24]. Our observations and those of others demonstrate that venoms of both adult and juvenile specimens exert thrombin-like (pseudo-procoagulant) activity. This activity has been previously described for adult *B*. *lanceolatus* venom [20,24]. This effect explains the weak procoagulant activity described for this venom on mouse citrated plasma. However, *in vitro* experiments do not necessarily reproduce what occurs *in vivo* and hence the importance of assessing hemostatic alterations *in vivo*.

We explored the *in vivo* effect of adult and juvenile venoms on classical clotting tests and rotational thromboelastometry parameters. No alterations were observed in PT and aPTT, and there was only a significant drop in the concentration of fibrinogen after i.v. injection. In contrast, when the venom of *B*. *asper*, which induces a typical consumption coagulopathy, was tested in a similar mouse model, there were drastic alterations in these parameters [46]. This agrees with clinical observations of *B*. *lanceolatus* envenomings since, in most cases, a consumption coagulopathy is not observed [3,5,11].

When *in vivo* effects were evaluated by rotational thromboelastometry, which provides a more in-depth assessment of the hemostatic status, the venom of juvenile specimens affected various Extem, Intem and Fibtem parameters 1 hr after i.v. injection, whereas only Fibtem parameters were altered in the case of adult venom. These findings might be due to the partial drop in fibrinogen concentration in mice injected with *B*. *lanceolatus* venoms as a consequence of the action of thrombin-like (pseudo-procoagulant) enzymes, since Fibtem basically depends on the status of fibrinogen. However, when venoms were administered i.p., in conditions where pulmonary thrombosis occurs, clotting tests were largely not altered, implying that thrombosis occurs in conditions where there is no defibrinogenation. The higher extent of rotational thromboelastometry alterations when using the i.v. route, as compared to the i.p. route, might be due to the fact that in the former the venom is in immediate contact with clotting factors in the bloodstream, thus increasing the likelihood of alterations. The lack of major alterations in rotational thromboelastometry parameters is in contrast with observations carried out with the venom of *B*. *asper*, which causes a consumption coagulopathy and drastically affects these parameters in a mouse model [46]. Overall, *B*. *lanceolatus* venoms do not induce *in vivo* consumption coagulopathy in this model, in agreement with clinical observations. The lack of overt consumption coagulopathy might be related to the thrombotic effect since clotting factors are present in the bloodstream by the time the thrombogenic mechanisms induced by the venom are operating in blood vessels. On the other hand, a drastic drop in platelet numbers was observed by the time thrombosis occurs, suggesting that platelet alterations might be related to the pathogenesis of thrombosis by mechanisms as yet unknown.

In conclusion, when injected by the i.p. route, the venom of juvenile specimens of *B*. *lanceolatus* induces abundant thrombi in the pulmonary vasculature, thus reproducing clinical findings describing thrombosis in patients bitten by snakes of small size in Martinique. The model also reproduces clinical observations describing thrombocytopenia and lack of consumption coagulopathy in many patients. This experimental model could be used to explore the mechanisms of thrombosis induced by the venom of *B*. *lanceolatus* and to identify the toxins responsible for this effect. Moreover, although rare, peripheral arterial thrombosis and pulmonary thromboembolism have been described in envenomings by other viperid snake species [47,48], thus opening the possibility of using this experimental model to explore the development of thrombosis with other snake venoms.

## Supporting information

S1 Table Details of the protein matches and supporting peptides related to the proteomic analysis of venoms of *B*. *lanceolatus* from adult and juvenile specimens. (XLSX)

S2 Table Raw data of the assays evaluating the alterations induced by the venoms of *B*. *lanceolatus* on classical clotting tests, rotational thromboelastometry, and platelet counts. (XLSX)

S3 Table Raw data of the histological assessment of thrombosis in histological sections from the lungs of mice receiving intraperitoneal injection of 70 μg of the venoms of juvenile and adult specimens of *B*. *lanceolatus*. (DOCX)

S1 Fig Comparison of the protein matches obtained by shotgun proteomic profiling of juvenile and adult venoms of *Bothrops lanceolatus* for (A) snake venom metalloproteinases; and (B) snake venom serine proteinases. Details of the matching venom proteins of the UniProt Serpentes database are presented in S1 Table.
(DOCX)

S2 Fig Representative Intem rotational thromboelastometry tracings from mice injected with PBS or venoms of juvenile and adult specimens of *B*. *lanceolatus*. Blood was collected by cardiac puncture under inhaled isoflurane anesthesia, added to sodium citrate solution, and evaluated by rotational thromboelastometry (see Methods for details). (A) Intem tracing from a mouse receiving PBS by the i.v. route and bled 1 hr after injection. (B) and (C) Intem tracings from mice receiving 20 μg of juvenile (B) or adult (C) *B*. *lanceolatus* venoms by the i.v. route and bled 1 hr after injection. (D) and (E) Intem tracings from mice receiving 70 μg of juvenile (D) or adult (E) venoms by the i.p. route and bled 4 hr after injection.
(JPG)

S3 Fig Representative Fibtem rotational thromboelastometry tracings from mice injected with PBS or venoms of juvenile and adult specimens of *B*. *lanceolatus*. Blood was collected by cardiac puncture under inhaled isoflurane anesthesia, added to sodium citrate solution, and evaluated by rotational thromboelastometry (see Methods for details). (A) Fibtem tracing from a mouse receiving PBS by the i.v. route and bled 1 hr after injection. (B) and (C) Fibtem tracings from mice receiving 20 μg of juvenile (B) or adult (C) *B*. *lanceolatus* venoms by the i.v. route and bled 1 hr after injection. (D) and (E) Fibtem tracings from mice receiving 70 μg of juvenile (D) or adult (E) venoms by the i.p. route and bled 4 hr after injection.
(JPG)

## References

[1] Wüster W, Thorpe RS, Salomao MG, Thomas L, Puorto G, Theakston RDG, et al. Origin and phylogenetic position of the Lesser Antillean species of *Bothrops* (Serpentes, Viperidae): biogeographical and medical implications. Bull Nat Hist Mus Lond (Zool) 2002; 68: 101–6.

[2] Thomas L, Tyburn B, and the Research Group of Snakebite in Martinique. *Bothrops lanceolatus* bites in Martinique. Clinical aspects and treatment. In: Bon C, Goyffon M, Editors. Envenomings and Their Treatment. Lyon: Fondation Marcel Mérieux; 1996. pp 255–65.

[3] Resiere D, Mégarbane B, Valentino R, Mehdaoui H, Thomas L. *Bothrops lanceolatus* bites: guidelines for severity assessment and emergent management. Toxins (Basel) 2010; 2: 163–73. doi: 10.3390/toxins2010163 22069552 PMC3206616

[4] Thomas L, Tyburn B, Bucher B, Pecout F, Ketterle J, Rieux D, et al. Prevention of thromboses in human patients with *Bothrops lanceolatus* envenoming in Martinique: failure of anticoagulants and efficacy of a monospecific antivenom. Am J Trop Med Hyg 1995; 52: 419–26. doi: 10.4269/ajtmh.1995.52.419 7771608

[5] Resiere D, Florentin J, Mehdaoui H, Kallel H, Legris-Alluson V, Gueye P, Neviere R. *Bothrops lanceolatus* envenoming in Martinique: A historical perspective of the clinical effectiveness of Bothrofav antivenom treatment. Toxins (Basel) 2024; 16: 146. doi: 10.3390/toxins16030146 38535812 PMC10976126

[6] Málaque CMS, Gutiérrez JM. Snakebite envenomation in Central and South America. In: Brendt et al, Editors. Critical Care Medicine. Switzerland: Springer International; 2016. pp 1–22.

[7] Otero-Patiño R., Epidemiological clinical and therapeutic aspects of *Bothrops asper* bites. Toxicon 2009; 54: 998–1011. doi: 10.1016/j.toxicon.2009.07.001 19591857

[8] Monteiro WM, Contreras-Bernal JC, Ferreira Bisneto P, Sachett J, Mendonca da Silva I, Lacerda M, et al. *Bothrops atrox*, the most important snake involved in human envenomings in the Amazon: How venomics contributes to the knowledge of snake biology and clinical toxicology. Toxicon:X 2020; 6: 100037. doi: 10.1016/j.toxcx.2020.100037 32550592 PMC7285970

[9] Houcke S, Puji JM, Vauquelin S, Ngoula GRL, Matheus S, NkontCho F, et al. Effect of time to antivenom administration on recovery from snakebite envenoming-related coagulopathy in French Guiana. PLoS Negl Trop Dis 2023; 17: e0011242. doi: 10.1371/journal.pntd.0011242 37093856 PMC10159357

[10] Thomas L, Tyburn B, Lang J, Ketterle J. Early infusion of a purified monospecific F(ab’)_2_ antivenom serum for *Bothrops lanceolatus* bites in Martinique. Lancet 1996; 347: 406. doi: 10.1016/s0140-6736(96)90590-5 8598738

[11] Thomas L, Tyburn B, Ketterlé J, Biao T, Mehdaoui H, Moravie V, et al. Prognostic significance of clinical grading of patients envenomed by *Bothrops lanceolatus* in Martinique. Trans R Soc Trop Med Hyg 1998; 92: 542–5. doi: 10.1016/s0035-9203(98)90907-5 9861375

[12] Malbranque S, Piercecchi-Marti MD, Thomas L, Barbey C, Coucier D, Bucher B, et al. Fatal diffuse thrombotic microangiopathy after a bite by the “Fer-de-Lance” pit viper (*Bothrops lanceolatus*) of Martinique. An J Trop Med Hyg 2008; 78: 856–61. 18541759

[13] Numeric P, Moravie V, Didier M, Chatot-Henry D, Cirille S, Bucher B, et al. Multiple cerebral infarctions following a snakebite by *Bothrops caribbaeus*. Am J Trop Med Hyg 2002; 67: 287–8. doi: 10.4269/ajtmh.2002.67.287 12408668

[14] Gutiérrez JM, Sanz L, Escolano J, Fernández J, Lomonte B, Angulo Y, et al. Snake venomics of the Lesser Antillean pit vipers *Bothrops caribbaeus* and *Bothrops lanceolatus*: correlation with toxicological activities and immunoreactivity of a heterologous antivenom. J Proteome Res 2008; 7: 4396–408. doi: 10.1021/pr8003826 18785768

[15] Pierre-Louis O, Resiere D, Alphonsine C, Dantin F, Banydeen R, Dubois MD, et al. Increased binding of von Willebrand factor to subendothelial collagen may facilitate thrombotic events complicating *Bothrops lanceolatus* envenomation in humans. Toxins (Basel) 2023; 15: 441. doi: 10.3390/toxins15070441 37505710 PMC10467054

[16] Delafontaine M, Villas-Boas I, Mathieu L, Josset P, Blomet J, Tambourgi D. Enzymatic and pro-inflammatory activities of *Bothrops lanceolatus* venom: relevance for envenomation. Toxins (Basel) 2017; 9: 244. doi: 10.3390/toxins9080244 28783135 PMC5577578

[17] Delafontaine M, Villas-Boas IM, Pidde G, van den Berg CW, Mathieu L, Blomet J, et al. Venom from *Bothrops lanceolatus*, a snake species native to Martinique, potently activates the complement system. J Immunol Res 2018; 2018: 3462136. doi: 10.1155/2018/3462136 30116749 PMC6079423

[18] Gabrili JJM, Pidde G, Magnoli FC, Marques-Puorto R, Villas-Boas IM, Squaiella-Baptistao CC, et al. New insights into immunopathology associated to *Bothrops lanceolatus* snake envenomation: focus on PLA_2_ toxin. Int J Mol Sci 2023; 24: 9931. doi: 10.3390/ijms24129931 37373079 PMC10298673

[19] Larréché S, Chevillard L, Jourdi G, Mathé S, Servonnet A, Joly BS, et al. *Bothrops* venom-induced hemostasis disorders in the rat: between Scylla and Charybdis. PLoS Negl Trop Dis 2023; 17: e0011786. doi: 10.1371/journal.pntd.0011786 38011218 PMC10703418

[20] Lobo de Araujo A, Donato JL, Bom C. Purification from *Bothrops lanceolatus* (der de lance) venom of a fibrino(geno)lytic enzyme with esterolytic activity. Toxicon 1998; 36: 745–58. doi: 10.1016/s0041-0101(97)00118-9 9655635

[21] Bogarín G., Romero M., Rojas G, Lutsch C, Casadamont M, Lang J, et al. Neutralization, by a monospecific *Bothrops lanceolatus* antivenom, of toxic activities induced by homologous and heterologous *Bothrops* snake venoms. Toxicon 1999; 37: 551–7. doi: 10.1016/s0041-0101(98)00193-7 10080358

[22] Guimaraes AQ, Cruz-Hofling MA, Ferreira de Araújo PM, Bon C, Lobo de Araújo A. Pharmacological and histopathological characterization of *Bothrops lanceolatus* (Fer de Lance) venom-induced edema. Inflamm Res 2004; 53: 284–91. doi: 10.1007/s00011-004-1258-0 15241562

[23] Bourke LA, Znedek CN, Tanaka-Azevedo AM, Silveira GPM, Sant’Anna SS, Grego KF, et al. Clinical and laboratory implications of dynamic coagulotoxicity. Divergences in *Bothrops* (lancehead pit viper) venoms. Toxins (Basel) 2022; 14: 297. doi: 10.3390/toxins14050297 35622544 PMC9148167

[24] Radouani F, Jalta P, Rapon C, Lexin C, Branford C, Florentin J, et al. The contrasting effects of *Bothrops lanceolatus* and *Bothrops atrox* venom on plasmatic procoagulant activity and thrombus stability under blood flow conditions. Toxins (Basel) 2024; 16: 400. doi: 10.3390/toxins/16090400 39330858 PMC11435654

[25] Bucher B, Canonge D, Thomas L, Tyburn B, Robbe-Vincent A, Choumet V, et al. Clinical indicators of envenoming and serum levels of venom antigens in patients bitten by *Bothrops lanceolatus* in Martinique. Trans R Soc Trop Med Hyg 1997; 91: 186–90. doi: 10.1016/s0035-9203(97)90219-4 9196765

[26] Lomonte B, Calvete JJ. Strategies in ‘snake venomics’ aiming at an integrative view of compositional, functional, and immunological characteristics of venoms. J Venom Anim Toxins Incl Trop Dis 2017; 23: 26. doi: 10.1186/s40409-017-0117-8 28465677 PMC5408369

[27] Lomonte B, Díaz C, Chaves F, Fernández J, Ruiz M, Salas M, et al. Comparative characterization of Viperidae snake venoms from Peru reveals two compositional patterns of phospholipase A_2_ expression. Toxicon:X 2020; 7: 100044. doi: 10.1016/j.toxcx.2020.100044 32550596 PMC7285926

[28] O’Leary MA, Isbister GK. A turbidimetric assay for the measurement of clotting times of procoagulant venoms in plasma. J Pharmacol Toxicol Methods 2010; 61: 27–31. doi: 10.1016/j.vascn.2009.06.004 19615454

[29] Sánchez A, Herrera M, Villalta M, Solano D, Segura Á, Lomonte B, et al. Proteomic and toxinological characterization of the venom of the South African ringhals cobra, *Hemachatus haemachatus*. J Proteomics 2018; 181: 104–17. doi: 10.1016/j.jprot.2018.04.007 29656017

[30] Resiere D, Arias AS, Villalta M, Rucavado A, Brouste Y, Cabié A, et al. Preclinical evaluation of the neutralizing ability of a monospecific antivenom for the treatment of envenomings by *Bothrops lanceolatus* in Martinique. Toxicon 2018; 148: 50.55. doi: 10.1016/j.toxicon.2018.04.010 29654867

[31] Rucavado A, Escalante T, Franceschi A, Chaves F, León G, Cury Y, et al. Inhibition of local hemorrhage and dermonecrosis induced by *Bothrops asper* snake venom: effectiveness of early in situ administration of the peptidomimetic metalloproteinase inhibitor batimastat and the chelating agent CaNa_2_EDTA. Am J Trop Med Hyg 2000; 63: 313–9. 11421384

[32] Shoyaib AA, Archie SR, Karamyan VT. Intraperitoneal route of drug administration: Should it be used in experimental animal studies? Pharm Res 2020; 37: 12. doi: 10.1007/s11095-019-2745-x 31873819 PMC7412579

[33] Bos LD, Ware LB. Acute respiratory distress syndrome: causes, pathophysiology and phenotypes. Lancet 2022; 400: 1145–56. doi: 10.1016/S0140-6736(22)01485-4 36070787

[34] Mitchell WB. Thromboinflammation in COVID-19 acute lung injury. Paediatric Resp Reviews 2020; 35: 20–24. doi: 10.1016/j.prrv.2020.06.004 32653469 PMC7289106

[35] Calvete JJ, Sanz L, Pérez A, Borges A, Vargas AM, Lomonte B, et al. Snake population venomics and antivenomics of *Bothrops atrox*: Paedomorphism along its transamazonian dispersal and implications of geographic venom variability on snakebite management. J Proteomics 2011; 74: 510–27. doi: 10.1016/j.jprot.2011.01.003 21278006

[36] Alape-Girón A, Sanz L, Escolano J, Flores-Díaz M, Madrigal M, Sasa M, et al. Snake venomics of the lancehead pitviper *Bothrops asper*: geographic, individual and ontogenetic variations. J Proteome Res 2008; 7: 3556–71. doi: 10.1021/pr800332p 18557640

[37] Warrell DA. Snakebites in Central and South America: Epidemiology, clinical features, and clinical management. In: Campbell JA, Lamar WW, editors. The Venomous Reptiles of the Western Hemisphere. Vol II. Ithaca: Comstock. pp. 709–61.

[38] Stroka A, Donato JL, Bon C, Hyslop S, Lobo de Araújo A. Purification and characterization of a hemorrhagic metalloproteinase from *Bothrops lanceolatus* (Fer-de-Lance) snake venom. Toxicon 2005; 45: 411–20. doi: 10.1016/j.toxicon.2004.11.010 15733562

[39] Terra RMS, Pinto AFM, Guimaraes JA, Fox JW. Proteomic profiling of snake venom metalloproteinases (SVMPs): insights into venom induced pathology. Toxicon 2009; 54: 836–44. doi: 10.1016/j.toxicon.2009.06.010 19539639

[40] Díaz C, Valverde L, Brenes O, Rucavado A, Gutiérrez JM. Characterization of events associated with apoptosis/anoikis induced by snake venom metalloproteinase BaP1 on human endothelial cells. J Cell Biochem 2005; 94: 520–8. doi: 10.1002/jcb.20322 15543558

[41] Gutiérrez JM, Rucavado A, Escalante T, Díaz C. Hemorrhage induced by snake venom metalloproteinases: biochemical and biophysical mechanisms involved in microvessel damage. Toxicon 2005; 45: 997–1011. doi: 10.1016/j.toxicon.2005.02.029 15922771

[42] Schattner M, Fritzen M, Ventura JS, de Albuquerque Modesto JC, Pozner G, Moura-da-Silva AM, et al. The snake venom metalloproteinases berythractivase and jararhagin activate endothelial cells. Biol Chem 2005; 386: 369–74. doi: 10.1515/BC.2005.044 15899699

[43] Teixeira C, Fernandes CM, Leiguez E, Chudzinski-Tavassi AM. Inflammation induced by platelet-activating snake venoms: perspectives on thromboinflammation. Front Immunol 2019; 10: 2082. doi: 10.3389/fimmu.2019.02082 31572356 PMC6737392

[44] Mandel J, Casari M, Stepanyan M, Martyanov, Deppermann C. Beyond hemostasis: platelet innate immune interactions and thromboinflammation. Int J Mol Sci 2022; 23: 3868. doi: 10.3390/ijms23073868 35409226 PMC8998935

[45] Herrera C, Rucavado A, Warrell DA, Gutiérrez JM. Systemic effects induced by the venom of the snake *Bothrops caribbaeus* in a murine model. Toxicon 2013; 63: 19–31. doi: 10.1016/j.toxicon.2012.10.023 23159397

[46] Rucavado A, Chacón M, Villalobos D, Arguello I, Campos M, Guerrero G, et al. Coagulopathy induced by viperid snake venoms in a murine model: Comparison of standard coagulation tests and rotational thromboelastometry. Toxicon 2022; 214: 121–9. doi: 10.1016/j.toxicon.2022.05.042 35644489

[47] Senthilkumaran S, Patel K, Rajan E, Vajayakumar P, Miller SW, Rucavado A, et al. Peripheral arterial thrombosis following Russell’s viper bites. TH Open 2023; 7: e168–83. doi: 10.1055/s-0043-1769625 37333023 PMC10276757

[48] Senthilkumaran S, Sampath S, Almeida JR, Williams J, Williams HF, Patel K, et al. Pulmonary thromboembolism following Russell’s viper bites. Toxins (Basel) 2024; 16: 222. doi: 10.3390/toxins16050222 38787074 PMC11125611

