# Supplementary material for: A murine experimental model of the pulmonary thrombotic effect induced by the venom of the snake Bothrops lanceolatus
Source: PLoS Negl Trop Dis. 2024 Oct 2;18(10):e0012335. doi: 10.1371/journal.pntd.0012335 (PMC11472959; doi:10.1371/journal.pntd.0012335)
Supplement: S3 Table — (DOCX) [file pntd.0012335.s003.docx]

**Supplementary Table S3**

**Analysis of thrombi formation in tissue sections from the lungs of mice injected with *B. lanceolatus* venoms (juvenile and adult specimens)**

The presence of thrombi was expressed as (+): presence of thrombi in blood vessels; (±): presence of hyaline material inside the blood vessel; (-) absence of thrombi and hyaline material inside blood vessels.

1. **Samples from mice injected i.p. with 70 µg venom from juvenile specimens**:

| Tissue section number | Presence of thrombi |
| --- | --- |
| 1 | + |
| 2 | + |
| 3 | - |
| 4 | + |
| 5 | + |
| 6 | + |
| 7 | + |
| 8 | + |
| 9 | + |
| 10 | + |
| 11 | - |
| 12 | + |
| 13 | + |
| 14 | - |
| 15 | + |
| 16 | + |
| 17 | ± |
| 18 | + |
| 19 | ± |
| 20 | + |
| 21 | + |
| 22 | + |
| 23 | ± |
| 24 | + |
| 25 | + |
| 26 | + |

1. **Samples from mice injected i.p. with 70 µg venom from adult specimens**

| Tissue section number | Presence of thrombi |
| --- | --- |
| 1 | ± |
| 2 | ± |
| 3 | - |
| 4 | - |
| 5 | - |
| 6 | ± |
| 7 | - |
| 8 | ± |
| 9 | - |
| 10 | - |
| 11 | ± |
| 12 | ± |
| 13 | + |
| 14 | - |
| 15 | - |
| 16 | - |

1. **Samples from mice injected i.p. with 70 µg venom from juvenile specimens preincubated with Batimastat**

| Tissue section number | Presence of thrombi |
| --- | --- |
| 1 | **+** |
| 2 | **+** |
| 3 | **-** |
| 4 | **+** |
| 5 | **+** |
| 6 | **+** |
| 7 | **+** |
| 8 | **±** |

1. **Samples from mice injected i.p. with 70 µg venom from adult specimens preincubated with Batimastat**

| Tissue section number | Presence of thrombi |
| --- | --- |
| **1** | **±** |
| **2** | **-** |
| **3** | **-** |
| **4** | **±** |
| **5** | **-** |
| **6** | **±** |
| **7** | **±** |
| **8** | **+** |
| **9** | **+** |
