# Supplementary material for: A murine experimental model of the pulmonary thrombotic effect induced by the venom of the snake Bothrops lanceolatus
Source: PLoS Negl Trop Dis. 2024 Oct 2;18(10):e0012335. doi: 10.1371/journal.pntd.0012335 (PMC11472959; doi:10.1371/journal.pntd.0012335)
Supplement: S1 Fig — Comparison of the protein matches obtained by shotgun proteomic profiling of juvenile and adult venoms of Bothrops lanceolatus for (A) snake venom metalloproteinases; and (B) snake venom serine proteinases. Details of the matching venom proteins of the UniProt Serpentes database are presented in S1 Table. (DOCX) [file pntd.0012335.s004.docx]

**(A)**


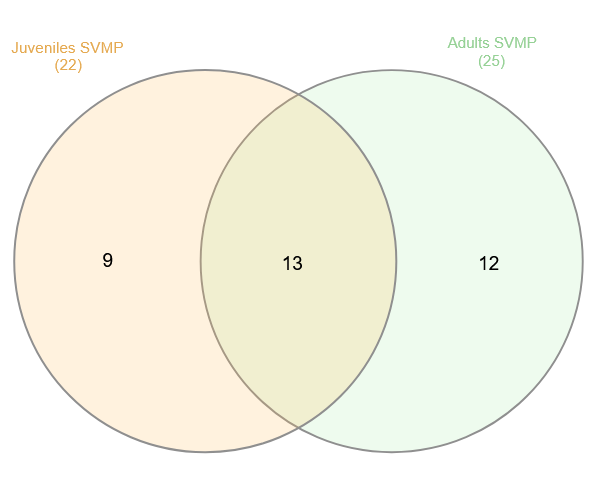


**(B)**


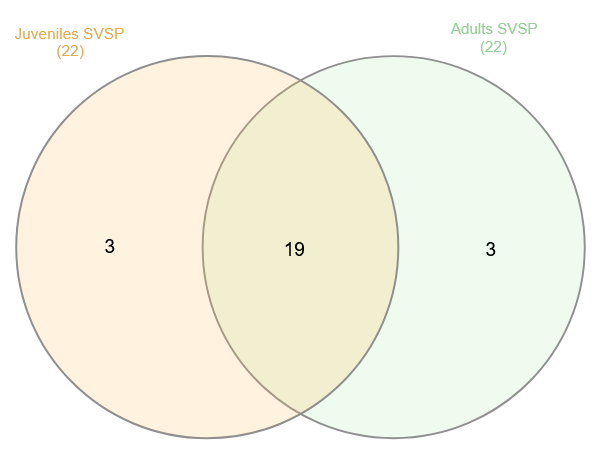


**Supplementary Figure S1:** Comparison of the protein matches obtained by shotgun proteomic profiling of juvenile and adult venoms of *Bothrops lanceolatus* for **(A)** snake venom metalloproteinases; and **(B)** snake venom serine proteinases. Details of the matching venom proteins of the UniProt Serpentes database are presented in Supplemenary Table S1.
