# Supplementary figures and images for: A murine experimental model of the pulmonary thrombotic effect induced by the venom of the snake Bothrops lanceolatus
Source: PLoS Negl Trop Dis. 2024 Oct 2;18(10):e0012335. doi: 10.1371/journal.pntd.0012335 (PMC11472959; doi:10.1371/journal.pntd.0012335)

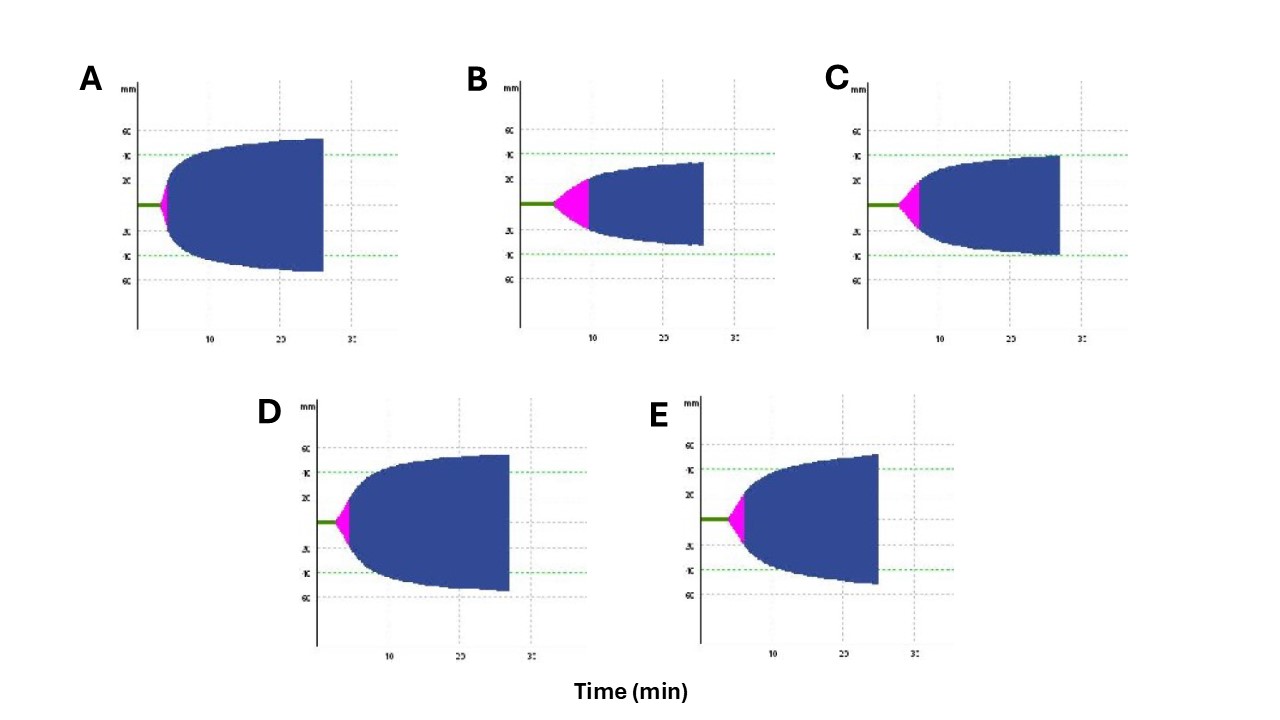

Supplement: S2 Fig — Representative Intem rotational thromboelastometry tracings from mice injected with PBS or venoms of juvenile and adult specimens of B. lanceolatus. Blood was collected by cardiac puncture under inhaled isoflurane anesthesia, added to sodium citrate solution, and evaluated by rotational thromboelastometry (see Methods for details). (A) Intem tracing from a mouse receiving PBS by the i.v. route and bled 1 hr after injection. (B) and (C) Intem tracings from mice receiving 20 μg of juvenile (B) or adult (C) B. lanceolatus venoms by the i.v. route and bled 1 hr after injection. (D) and (E) Intem tracings from mice receiving 70 μg of juvenile (D) or adult (E) venoms by the i.p. route and bled 4 hr after injection. (JPG) [file pntd.0012335.s005.jpg]

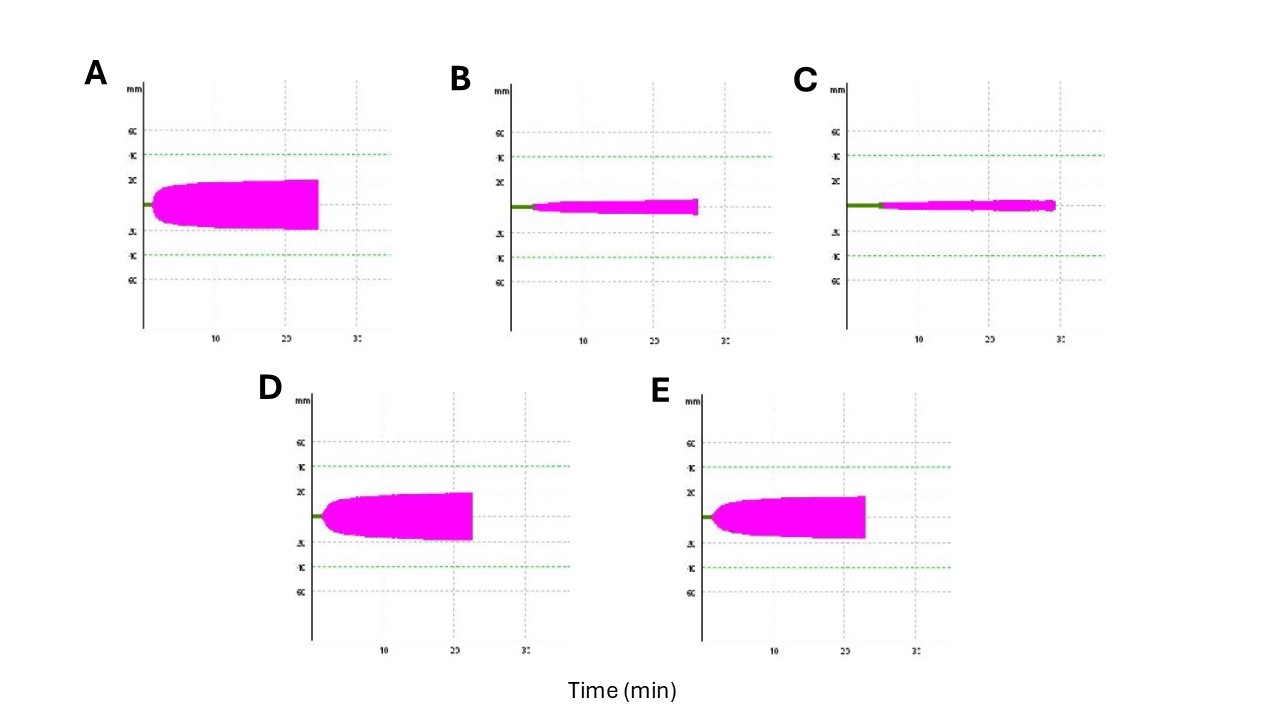

Supplement: S3 Fig — Representative Fibtem rotational thromboelastometry tracings from mice injected with PBS or venoms of juvenile and adult specimens of B. lanceolatus. Blood was collected by cardiac puncture under inhaled isoflurane anesthesia, added to sodium citrate solution, and evaluated by rotational thromboelastometry (see Methods for details). (A) Fibtem tracing from a mouse receiving PBS by the i.v. route and bled 1 hr after injection. (B) and (C) Fibtem tracings from mice receiving 20 μg of juvenile (B) or adult (C) B. lanceolatus venoms by the i.v. route and bled 1 hr after injection. (D) and (E) Fibtem tracings from mice receiving 70 μg of juvenile (D) or adult (E) venoms by the i.p. route and bled 4 hr after injection. (JPG) [file pntd.0012335.s006.jpg]
